# First-principles discovery of multifunctional K_2_NaRhX_6_ (X = F, Cl, Br, I) halide double perovskites for photovoltaic and photocatalytic applications

**DOI:** 10.1039/d6ra07563k

**Published:** 2026-09-30

**Authors:** Imtiaz Ahamed Apon, Gargee Mollika Suchi, Rifat Rafiu, Md. Azizur Rahman, Samar O. Aljazzar, Jehan Y. Al-Humaidi, Mohamed Benghanem, Safwat A. Mahmoud

**Affiliations:** a Electronics and Information Technology, University of South Wales Treforest Pontypridd CF37 1DL UK; b Department of Electrical and Electronic Engineering, Bangladesh Army University of Science and Technology (BAUST) Saidpur-5311 Bangladesh; c Department of Material Science and Engineering, Khulna University of Engineering & Technology (KUET) Khulna-9203 Bangladesh; d Department of Electrical and Electronic Engineering, Begum Rokeya University Rangpur 5400 Bangladesh azizurrahmanatik49@gmail.com; e Department of Chemistry, College of Science, Princess Nourah bint Abdulrahman University P.O. Box 84428 Riyadh 11671 Saudi Arabia; f Physics Department, Faculty of Science, Islamic University of Madinah Madinah 42351 Saudi Arabia; g Center for Scientific Research and Entrepreneurship, Northern Border University Arar 73213 Saudi Arabia

## Abstract

Lead-free halide double perovskites have emerged as attractive multifunctional materials for sustainable energy conversion owing to their compositional flexibility and tunable electronic structures. Herein, a comprehensive first-principles investigation combined with SCAPS-1D device simulations is performed to explore the structural stability, electronic structure, optical response, mechanical behavior, photocatalytic activity, and photovoltaic performance of K_2_NaRhX_6_ (X = F, Cl, Br, I). All compounds crystallize in the cubic *Fm*3̄*m* phase and exhibit negative formation enthalpies relative to their constituent elemental reference states. Halogen substitution systematically modulates the Rh-4d/X-p orbital hybridization, leading to a continuous reduction in the direct band gap from 1.861 to 0.579 eV together with enhanced visible-light absorption and dielectric response. The Br- and I-based compounds exhibit comparatively softer and more ductile mechanical characteristics favorable for thin-film processing, while calculated band-edge positions indicate suitable redox alignment for solar-driven water splitting. DFT-derived electronic parameters were subsequently employed in SCAPS-1D simulations of FTO/SnS_2_/K_2_NaRhX_6_ solar cells. After optimizing the absorber thickness, acceptor concentration, and defect density, K_2_NaRhCl_6_ delivered the highest theoretical photovoltaic performance with a power conversion efficiency of 26.64%, together with a *V*_OC_ of 0.918 V, *J*_SC_ of 34.89 mA cm^−2^, and FF of 83.23%. These findings demonstrate that halogen engineering effectively tailors the multifunctional properties of K_2_NaRhX_6_ double perovskites and identify K_2_NaRhCl_6_ and K_2_NaRhBr_6_ as promising lead-free candidates for next-generation photovoltaic and photocatalytic energy-conversion technologies.

## Abstract 2

Lead-free halide double perovskites have emerged as attractive multifunctional materials for sustainable energy conversion owing to their compositional flexibility and tunable electronic structures.

## Graphical abstract

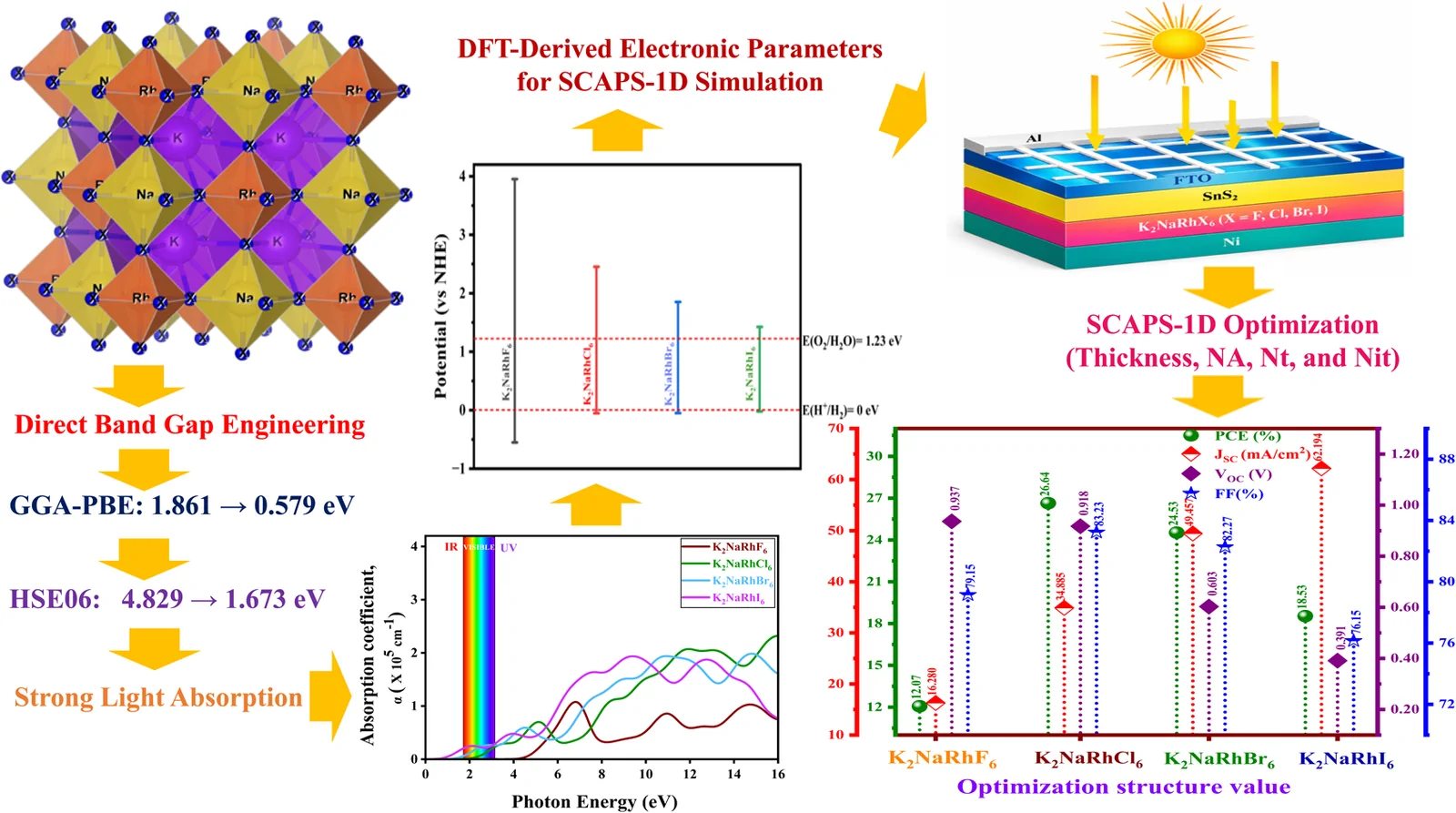

## 1. Introduction

The demand for sustainable energy technologies is growing day by day. It has intensified the search for absorbers and photocatalytic materials. Particularly, materials that combine chemical stability, non-toxicity, and suitable optoelectronic characteristics are at the peak of interest.^1–3^ Hybrid lead-halide perovskites are well known for their higher power conversion efficiencies comparable to conventional semiconductor technologies.^4,5^ But there are some concerns, including Pb toxicity, moisture sensitivity, and thermal instability, that continue to limit their long-term applicability.^6^ These issues have motivated extensive investigation of lead-free inorganic perovskites, particularly double perovskites with the general chemical formula A_2_BB′X_6_. In contrast to conventional ABX_3_ single perovskites, double perovskites offer broader compositional flexibility and improved structural stability due to ordered occupation of the B and B′ cation sites.^7,8^ Their electronic structures can be tuned through cation substitution, halogen engineering, and lattice modification, which makes them relevant for photovoltaic conversion, photocatalytic water splitting, photodetection, thermoelectric devices, and ultraviolet optoelectronics.^9–12^ In many cases, the optical response and transport behavior are strongly governed by the hybridization between transition-metal d-states and halogen p-orbitals, while structural stability is linked to octahedral connectivity, tolerance factors, and elastic behavior within the cubic lattice.^13,14^

Recent density functional theory (DFT) studies suggest that several lead-free double perovskites exhibit electronic and optical characteristics compatible with solar-energy conversion. Vacancy-ordered A_2_BX_6_-type double perovskites have attracted increasing attention because of their tunable band gaps, strong optical absorption, defect tolerance, and comparatively improved structural stability relative to conventional Pb-based perovskites. The electronic and photovoltaic behavior of these systems is strongly influenced by A-site and halogen substitution, which modifies lattice expansion, orbital hybridization, and carrier transport properties. Recent studies on vacancy-ordered double perovskites such as A_2_CeCl_6_ (A = K, Rb, Cs, Fr), D_2_CeX_6_ (D = In, Ga, Tl; X = Cl, Br), A_2_GeX_6_ (A = K, Rb; X = Cl, Br), In_2_GeX_6_ (X = Cl, Br), and Cs_2_TiBr_6_ have attracted considerable attention for photovoltaic applications.^15–19^ These compounds were reported to possess suitable band gaps, strong ultraviolet-visible optical absorption, and good mechanical and thermal stability. SCAPS-1D simulations further suggested that several of these materials can achieve favorable photovoltaic performance in lead-free solar-cell architectures. Alongside vacancy-ordered systems, conventional A_2_BB′X_6_ double perovskites have also received considerable attention due to their chemically ordered octahedral framework, flexible electronic structure, and enhanced compositional tunability for photovoltaic and optoelectronic applications. Similar behavior has been reported for Cs_2_AgBiBr_6_ and Na_2_AgBiBr_6_, where the calculated band gaps, optical absorption spectra, and dielectric response indicate semiconducting behavior suitable for photovoltaic applications.^20^ In these systems, Ag-d and halogen-p hybridization plays a major role in defining the valence-band topology and carrier transport characteristics. Mechanical stability and thermodynamic formability, evaluated through elastic constants and negative formation energies, were also found to support experimental feasibility. Related investigations on Cs_2_NaInX_6_ (X = Cl, Br, I) demonstrated that halogen substitution substantially modifies the band gap and optical absorption edge through lattice expansion and orbital-energy shifts.^21^ A broader computational screening based on machine-learning-assisted DFT calculations further identified K_2_NaInI_6_ and Na_2_MgMnI_6_ as potentially stable semiconductors with photovoltaic band gaps comparable to those of methylammonium lead iodide.^22^ Beyond photovoltaic applications, several double perovskites have also been examined for thermoelectric and optoelectronic behavior, including Cs_2_AgXBr_6_, ABiCuX_6_, and related halide compounds, where moderate band gaps, high optical conductivity, and low reflectivity were associated with enhanced visible-light response and improved carrier dynamics.^23,24^ Additional studies on Cs_2_AgXBr_6_, Cs_2_AgBiBr_6_, and Cs_2_AgSbBr_6_ further demonstrated desirable electronic structures, strong visible-light absorption, thermoelectric behavior, and photovoltaic suitability under different theoretical approaches.^23,25^ More recently, Rh-containing fluoride double perovskites A_2_AgRhF_6_ (A = K, Rb, Cs) were investigated using combined DFT and SCAPS simulations.^26^ Those studies indicated that lattice expansion induced by larger A-site cations modifies the d–p hybridization strength and electronic dispersion, thereby affecting both optical absorption and photovoltaic performance.

Parallel to these photovoltaic studies, increasing attention has been directed toward photocatalytic applications of double perovskites, particularly for solar-driven hydrogen evolution. The feasibility of photocatalytic water splitting is generally determined by the relative positions of the conduction-band minimum (CBM) and valence-band maximum (VBM) with respect to the water redox potential.^27^ In this regard, compounds such as Rb_2_SnBr_6_, Tl_2_BPI_6_, and Sr_2_BiXO_6_ were reported to exhibit energetically favorable band-edge alignment for hydrogen and oxygen evolution reactions.^28–30^ Their photocatalytic response is often accompanied by strong ultraviolet-visible absorption and moderate band gaps that facilitate solar-light utilization. Investigations on Ba_2_XSbO_6_ and Cs_2_BB′H_6_ also suggested that elastic stability, negative formation energies, and tunable electronic structures are closely linked to photocatalytic efficiency.^31,32^ In several cases, Mulliken or Hirshfeld population analyses revealed substantial charge redistribution between cationic and anionic sublattices, implying mixed ionic-covalent bonding characteristics that may influence carrier separation and recombination dynamics during photocatalytic processes. Nevertheless, despite the growing number of studies on lead-free double perovskites, Rh-based halide systems remain comparatively less explored, especially regarding the combined relationship between bonding characteristics, band-edge alignment, photocatalytic activity, and device-level photovoltaic performance. The selection of Rh as the B-site cation is motivated by the ability of its 4d orbitals to interact with halogen p orbitals, which can provide an additional degree of tunability in the electronic structure and optical response. Compared with several extensively studied Ag-, Bi-, and In-based double perovskites, Rh-based systems remain less explored and may offer distinct electronic and bonding characteristics arising from Rh–X orbital hybridization. These features make Rh-based double perovskites promising candidates for investigating tunable optoelectronic and photocatalytic properties.

Motivated by these considerations, the present work examines the multifunctional properties of K_2_NaRhX_6_ (X = F, Cl, Br, I) double perovskites through first-principles DFT calculations combined with SCAPS-1D device simulations. The study focuses on the interplay between structural stability, electronic structure, optical absorption, elastic behavior, and energy-conversion performance. Attention is given to the effect of halogen substitution on lattice expansion, d–p orbital hybridization, and band-gap evolution. Mulliken and Hirshfeld population analyses are employed to investigate charge transfer and bonding behavior, while electronegativity-based band-edge calculations are used to evaluate photocatalytic suitability relative to water oxidation and reduction potentials. In addition, SCAPS simulations are performed to assess photovoltaic parameters and absorber-layer performance under realistic device conditions. By combining atomistic electronic analysis with photocatalytic and photovoltaic evaluation in a single framework, this work aims to provide a more complete understanding of Rh-based lead-free double perovskites for renewable-energy applications.

## 2. Computational method

### 2.1. CASTEP simulations

First-principles calculations were carried out within the framework of density functional theory (DFT) using a plane-wave pseudopotential approach.^33,34^ The K_2_NaRhX_6_ (X = F, Cl, Br, I) compounds were constructed based on the conventional cubic A_2_BB′X_6_ double-perovskite prototype (space group *Fm*3̄*m*). K occupies the A site, while Na and Rh occupy the ordered B and B′ sites, respectively. The initial structures were fully optimized by relaxing both lattice parameters and atomic positions until the convergence criteria were satisfied. Halogen substitution (F, Cl, Br, and I) was then employed to systematically investigate its effect on the structural and optoelectronic properties. The exchange–correlation interaction was treated using the generalized gradient approximation (GGA) in the Perdew–Burke–Ernzerhof (PBE) functional.^35–37^ To overcome the well-known underestimation of band gaps in semi-local functionals, selected electronic-structure calculations were further refined using the hybrid HSE06 functional, which incorporates a fraction of screened Hartree–Fock exchange.^38,39^ A plane-wave pseudopotential approach was employed to model the electron–ion interactions, with relativistic effects incorporated through the Koelling–Harmon approximation. Geometry optimizations were performed using stringent convergence criteria, including an energy tolerance of 1.0 × 10^−5^ eV per atom, a maximum force threshold of 0.03 eV Å^−1^, a maximum stress of 0.05 GPa, and a maximum atomic displacement of 0.001 Å, ensuring accurate determination of the equilibrium crystal structures. The plane-wave cut-off energy was set to 520 eV, which was found to be sufficient for achieving convergence of total energies and interatomic forces. Self-consistent field (SCF) calculations were carried out using an ultrafine accuracy setting to obtain reliable electronic properties. Structural optimization was performed using the Broyden–Fletcher–Goldfarb–Shanno (BFGS) minimization algorithm, whereby the atomic coordinates and lattice parameters were iteratively relaxed until the Hellmann–Feynman forces reached their convergence limits. Brillouin-zone integrations were conducted using a Monkhorst–Pack *k*-point mesh of 5 × 5 × 5, providing adequate sampling for the calculation of electronic, elastic, and optical properties. Phonon calculations were performed within the framework of density-functional perturbation theory (DFPT) to investigate the lattice dynamics of the optimized K_2_NaRhX_6_ structures. The dynamical matrices were evaluated on a 2 × 2 × 2 *q*-point grid, corresponding to force constants obtained from a 2 × 2 × 2 supercell. The resulting force constants were Fourier-interpolated along the selected high-symmetry paths to obtain the phonon dispersion relations. The phonon calculations were performed using the optimized structural models obtained from the preceding geometry-relaxation procedure. The valence electron configurations considered in the calculations were K (3p^6^4s^1^), Na (2p^6^3s^1^), Rh (4d^8^5s^1^), F (2s^2^2p^5^), Cl (3s^2^3p^5^), Br (4s^2^4p^5^), and I (5s^2^5p^5^). These electronic configurations ensured an accurate representation of the valence states in the K_2_NaRhX_6_ (X = F, Cl, Br, I) compounds. Within this computational framework, the structural, electronic, elastic, and optical properties of the investigated double perovskites were systematically examined. The calculated properties included equilibrium lattice parameters, formation energies, electronic band structures, total and partial density of states (DOS and PDOS), phonon dispersion relations, elastic constants and derived mechanical parameters, as well as optical characteristics such as the dielectric function, absorption coefficient, reflectivity, refractive index, energy-loss function, and optical conductivity. These calculations provide a comprehensive understanding of the structural, electronic, lattice-dynamical, and optical properties of the K_2_NaRhX_6_ (X = F, Cl, Br, I) series.

### 2.2. SCAPS-1D simulations

The solar cell simulations were performed using the SCAPS-1D simulator under standard AM 1.5G illumination (100 mW cm^−2^) at 300 K. The device structure consisted of FTO/CdS/K_2_NaRhX_6_/HTL, where X = F, Cl, Br, and I. SCAPS-1D solved Poisson's and carrier continuity equations to analyze the photovoltaic performance.^40,41^ The electronic and optical properties of the K_2_NaRhX_6_ absorber layers, including bandgap, electron affinity, dielectric permittivity, effective density of states, and carrier transport parameters, were extracted from the density functional theory (DFT) calculations performed in this work. The material parameters of the FTO and SnS_2_ layers were adopted from previously reported theoretical and experimental studies. The initial device configuration and input parameters used in the SCAPS-1D simulations are summarized in Table S1. To achieve optimal device performance, key parameters such as absorber-layer thickness, bulk defect density, interface defect density, and shallow acceptor concentration were systematically varied and optimized. The optimization process was carried out by evaluating the corresponding changes in the open-circuit voltage (*V*_OC_), short-circuit current density (*J*_SC_), fill factor (FF), and power conversion efficiency (PCE). The optimized parameter set was subsequently employed to assess the photovoltaic potential of the investigated K_2_NaRhX_6_ absorber materials.

## 3. Analysis and discussion of results using CASTEP

### 3.1. Simulated X-ray diffraction (XRD) patterns

The crystal structures of K_2_NaRhX_6_ (X = F, Cl, Br, I) were investigated using theoretical X-ray diffraction (XRD) patterns simulated *via* the CASTEP code within density functional theory. Fig. 1 presents the simulated XRD patterns of the investigated compounds. The diffraction spectra exhibit distinct reflections indexed to the (111), (200), (220), (311), (222), (400), (331), (420), and (422)/(510) crystallographic planes, indicating well-ordered crystal structures. The sharp diffraction peaks and the absence of significant peak broadening suggest a high degree of crystallographic ordering in the optimized models.

**Fig. 1:**
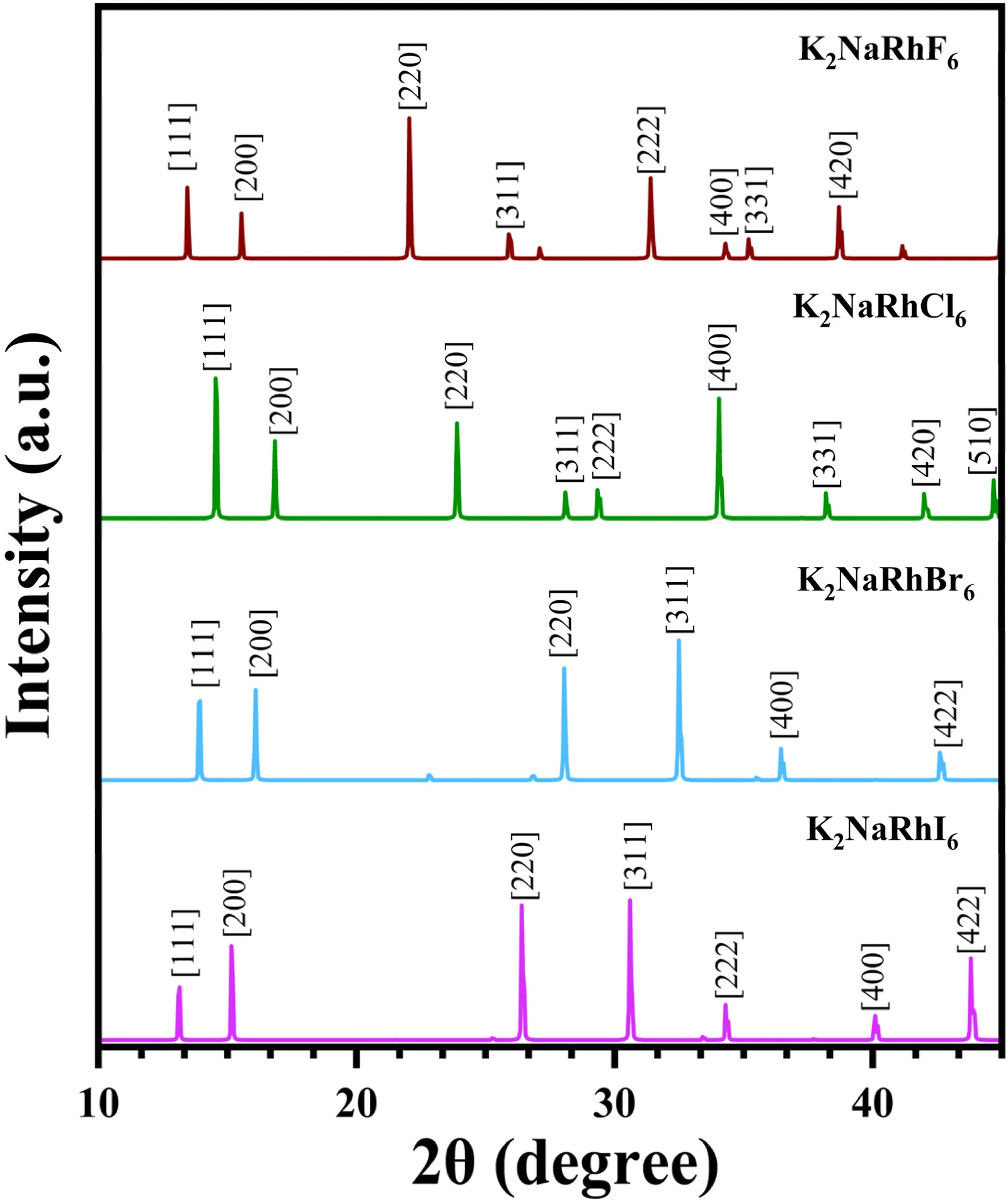
X-ray diffraction (XRD) patterns of K_2_NaRhX_6_ (where X = F, Cl, Br, I) compounds showing the effect of X-site anion size on crystal structure and lattice expansion.

The simulated diffraction patterns are consistent with the optimized cubic *Fm*3̄*m* structural models and serve as theoretical reference patterns for future experimental characterization. The preservation of similar diffraction features across the halogen series confirms that the cubic crystal structure remains stable upon substitution of F, Cl, Br, and I at the X site.

A systematic shift of diffraction peaks toward lower 2*θ* values is observed from K_2_NaRhF_6_ to K_2_NaRhI_6_, which can be attributed to the progressive increase in halogen ionic radius. The larger anionic size expands the RhX_6_ octahedra and increases the lattice volume, thereby enlarging the interplanar spacing. This trend is fully consistent with Bragg's law (*nλ* = 2*d* sin *θ*), where larger interplanar spacings result in smaller diffraction angles.^42^ The optimized lattice parameters of 6.111, 7.233, 7.694, and 8.340 Å for K_2_NaRhF_6_, K_2_NaRhCl_6_, K_2_NaRhBr_6_, and K_2_NaRhI_6_, respectively, yield diffraction peak positions consistent with the optimized crystal structures and reflect the systematic lattice expansion caused by halogen substitution. It should be noted that these XRD patterns are theoretically generated from the optimized crystal structures and therefore represent predicted diffraction patterns that may serve as reference data for future experimental studies. Experimental factors such as defects, strain, preferred orientation, and secondary phases are not considered in the present simulations and would require experimental XRD measurements for comprehensive verification.

### 3.2. Structural properties analysis

The investigated K_2_NaRhX_6_ (X = F, Cl, Br, I) compounds crystallize in the cubic double-perovskite structure with space group *Fm*3̄*m* (no. 225). In this structure (Fig. 2), Rh atoms occupy the 4a site (0, 0, 0) that forms RhX_6_ octahedra and Na atoms occupy the 4b site (0, 0, 1/4) forming NaX_6_ octahedra. K atoms are located at the 8c position (1/4, 1/4, 3/4) and halogen atoms occupy the-24e sites (*x*, 0, 0).

**Fig. 2:**
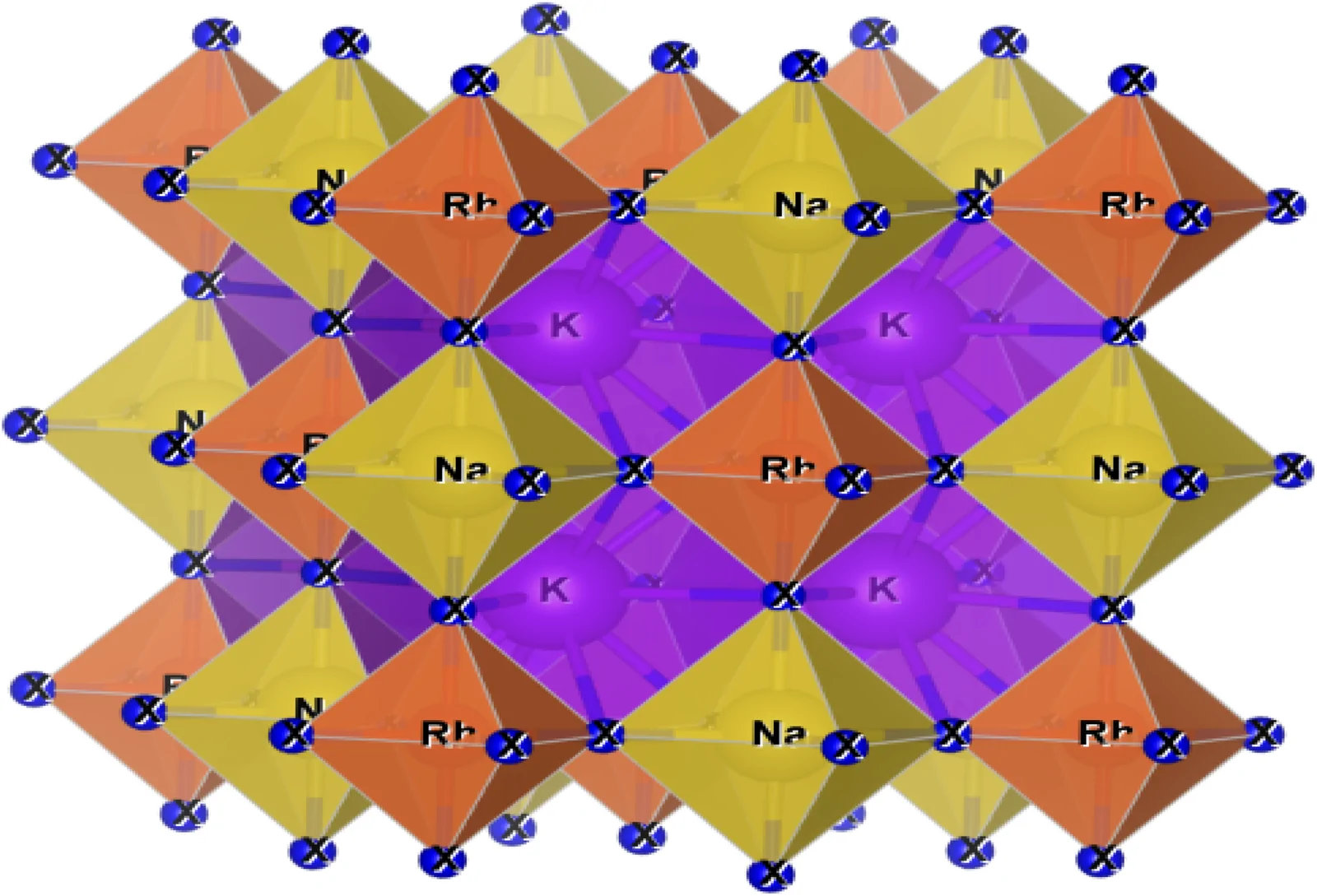
Crystal structure of K_2_NaRhX_6_ double perovskite materials.

The corner-sharing RhX_6_ and NaX_6_ octahedra generate a highly symmetric three-dimensional framework, indicating structural robustness.

The thermodynamic stability of the double perovskite has been assessed by its formation energy calculated using the formula below:^43^

1 Δ*E*_f_ = *E*_tot_(K_2_NaRhX_6_) − 2*E*(K) − *E*(Na) − *E*(Rh) − 6*E*(X)

where *E*_tot_(K_2_NaRhX_6_) is the total energy of the compound and *E*(K), *E*(Na), *E*(Rh), and E(X) represent the energies of the constituent elements in their reference states. The calculated lattice constants increase systematically from 6.111 Å for K_2_NaRhF_6_ to 8.340 Å for K_2_NaRhI_6_, while the GGA-PBE band gap decreases from 1.861 to 0.579 eV with increasing halide size. These systematic trends provide a useful basis for comparison with previously reported Rh-based and related double perovskites.^44^ As presented in Table 1, the calculated formation enthalpies are negative for all investigated compounds, with values of −4.712, −3.635, −3.266, and −2.845 eV per atom for K_2_NaRhF_6_, K_2_NaRhCl_6_, K_2_NaRhBr_6_, and K_2_NaRhI_6_, respectively. These negative values indicate that the formation of the investigated compounds from their constituent elemental reference states is energetically favorable. However, formation enthalpy alone does not establish thermodynamic stability against decomposition into competing K–Na–Rh–X phases. The gradual decrease in the magnitude of the formation enthalpy from F to I suggests a reduction in the energetic stabilization across the halide series. Among the investigated compounds, K_2_NaRhF_6_ exhibits the most negative formation enthalpy, indicating the strongest energetic stabilization relative to the constituent elemental reference states within the present series.

**Table 1:** Energy band gap, lattice constants, unit cell volume and formation enthalpy of K_2_NaRhX_6_ double perovskites

Compounds	GGA-PBE				Hybrid-HSE06		Formation enthalpy, ^ΔH^*f* (eV per atom)
	Energy band gap, eV	Lattice constant *a* (Å)	Unit cell volume, *V* (Å^3^)	Density, g cm^−1−3^	Energy band gap, eV	Unit cell volume, *V* (Å^3^)	
K_2_NaRhF_6_	1.861	6.111	161.448	3.271	4.829	161.477	−4.712
K_2_NaRhCl_6_	1.267	7.233	267.679	2.585	3.204	269.307	−3.635
K_2_NaRhBr_6_	0.936	7.694	322.088	3.523	2.660	322.090	−3.266
K_2_NaRhI_6_	0.579	8.340	410.311	3.907	1.673	410.321	−2.845

From a chemical perspective, the K_2_NaRhX_6_ (X = F, Cl, Br, I) family provides a chemically tunable platform for investigating the influence of halide substitution on the structural, energetic, bonding, and electronic characteristics of Rh-based double perovskites. The systematic variation of lattice parameters, formation enthalpies, and electronic properties across the halide series provides an opportunity to establish composition–structure–property relationships within a common crystallographic framework. Such chemical tunability is particularly relevant for understanding how the nature of the halide anion influences the stability and functional properties of transition-metal halide double perovskites.

### 3.3. Proposed synthesis routes and experimental validation

The preparative reaction proposed here is 2 KX(s) + NaX(s) + RhX_3_(s) → K_2_NaRhX_6_(s). It should be noted that K_2_NaRhF_6_ is already known experimentally: Wilhelm and Hoppe obtained it, together with Rb_2_NaRhF_6_ and Cs_2_KRhF_6_, as pink cubic elpasolite powders,^45^ in good agreement with the present structural calculations. The heavier members of the series remain unreported. A single procedure cannot serve all four halides, because they differ markedly in oxidizing power, with standard reduction potentials falling from +2.87 V for fluorine to +0.54 V for iodine, and in the solubility and hydrolytic stability of their Rh(iii) precursors. All solution routes are further limited by the kinetic inertness of the low-spin d^6^ Rh(iii) centre, which requires longer reaction times than the Ag(i)- and Bi(iii)-based double perovskites. For the fluoride, aqueous HF routes are appropriate, as established for the isostructural K_2_NaAlF_6_ and K_2_LiAlF_6_;^46^ high-temperature fluorination must be avoided, since RhF_3_ is converted to RhF_5_ above about 673 K. For the chloride and the bromide, a hydrothermal route analogous to that used for Cs_2_NaBCl_6_ elpasolites is proposed: RhX_3_·*x*H_2_O, NaX and KX in a 1 : 1 : 2 molar ratio in 10 mL of concentrated HX (12 M HCl or 8.8 M HBr, a three-fold excess suppressing hydrolysis), held in a PTFE-lined autoclave at 453 K for 12 to 48 h under autogenous pressure and cooled at 2 to 5 K per hour to grow single crystals;^47^ bromide reactions require an inert atmosphere.

The iodide is the most demanding member, consistent with its least negative formation enthalpy and with the reported instability of iodide elpasolites towards competing phases.^48^ Kinetically controlled routes are therefore preferred: sealed evacuated silica ampoules at 573 to 673 K for 24 to 48 h,^49^ or mechanochemical milling at 400 to 600 rpm followed by annealing at 523 K.^50^ In solution, 57% HI stabilized with 1 to 2 vol% H_3_PO_2_ suppresses I_2_ formation.^51^ The most likely competing phases are KX, NaX, RhX_3_, A_3_RhX_6_ and A_3_Rh_2_X_9_. Notably, Cs_2_NaRhCl_6_ adopts a 12R face-sharing perovskite rather than the cubic elpasolite structure,^52^ so the cubic assumption should be confirmed experimentally for the heavier halides by Rietveld refinement in *Fm*3̄*m* against the simulated patterns of Fig. 1, supported by SEM-EDS, XPS, TGA-DSC and UV-vis diffuse-reflectance measurements.

### 3.4. Geometrical stability parameters

The geometrical stability of double-perovskite can be assessed using the Goldschmidt tolerance factor (*t*) and octahedral factor (*µ*), which provide insight into the structural compatibility of constituent ions within the double-perovskite framework. The tolerance factor, defined as,2
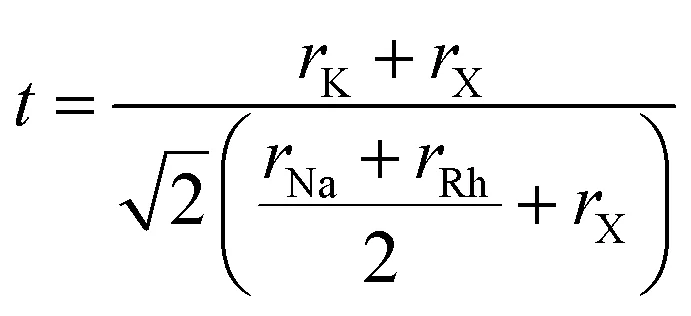
depends on the ionic radii of the A-site cation (*r*_A_), B-site cation (*r*_B_), and halide anion (*r*_X_). For most stable perovskite and double-perovskite structures, the tolerance factor generally lies between 0.8 and 1.0. The octahedral factor, expressed as,^53^3
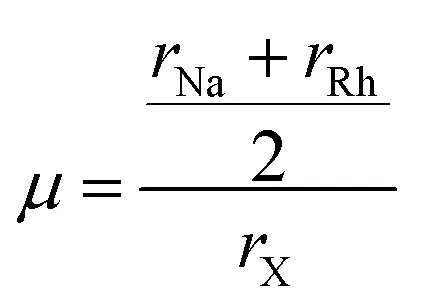
which describes the size compatibility between the B-site cations and the surrounding halide ions within the octahedral environment.^54,55^ The calculated values of (*t*) and (*µ*) for K_2_NaRhX_6_ (X = F, Cl, Br, I) are listed in Table 2. The tolerance factor decreases gradually from 0.966 for K_2_NaRhF_6_ to 0.892 for K_2_NaRhI_6_ as the ionic radius of the halogen increases from F^−^ to I^−^. Nevertheless, all values remain within the accepted stability range for perovskite materials, indicating that the double-perovskite framework is geometrically favorable. The value of (*t*) closest to unity for K_2_NaRhF_6_ suggests a nearly ideal cubic structure, while the progressive reduction in (*t*) for the chloride, bromide, and iodide compounds indicates a gradual departure from ideal ionic packing. A similar decreasing trend is observed for the octahedral factor, which declines from 0.633 to 0.383 with increasing halogen size.

**Table 2:** Tolerance factor variation in K_2_NaRhX_6_ double perovskite

Materials	*r*_K_	*r*_Na_	*r*_Rh_	*r*_F_	Tolerance factor (*t*)	Octahedral factor (*µ*)
K_2_NaRhF_6_	1.64	1.02	0.665	1.33	0.966	0.633
K_2_NaRhCl_6_	1.64	1.02	0.665	1.81	0.919	0.465
K_2_NaRhBr_6_	1.64	1.02	0.665	1.96	0.908	0.429
K_2_NaRhI_6_	1.64	1.02	0.665	2.2	0.892	0.383

This behavior reflects the increasing dimensions of the halide octahedra and the resulting geometric mismatch between the B-site cations and surrounding anions. Although the octahedral factor of K_2_NaRhI_6_ is slightly lower than the conventional empirical limit, the optimized structure retains the cubic *Fm*3̄*m* symmetry and exhibits a negative formation enthalpy. These results support the geometric plausibility of the proposed double-perovskite framework, although they do not by themselves establish complete dynamical or thermodynamic stability. The simultaneous decrease in both *t* and *µ* is consistent with the increase in lattice constants and unit-cell volumes from fluoride to iodide. Overall, the calculated geometrical parameters indicate that the investigated K_2_NaRhX_6_ compounds are geometrically compatible with the double-perovskite framework.

### 3.5. Electronic band structures

The calculated electronic band structures of K_2_NaRhX_6_ (X = F, Cl, Br, I) double perovskites obtained using the GGA-PBE and HSE06 functionals are presented in Fig. 3(a) and (b), respectively. In both figures, the valence band maximum (VBM) is located below the Fermi level (0 eV), while the conduction band minimum (CBM) lies above it. The electronic dispersion is plotted along the high-symmetry directions *W*–*L*–*Γ*–*X*–*W*–*K* of the first Brillouin zone. A careful examination of the band structures reveals that both the VBM and CBM occur at the *Γ* point for all investigated compounds. This confirms the direct band-gap nature of K_2_NaRhX_6_. The calculated band-gap values are summarized in Table 1. Using the GGA-PBE functional, the band gaps are found to be 1.861 eV, 1.267 eV, 0.936 eV, and 0.579 eV for K_2_NaRhF_6_, K_2_NaRhCl_6_, K_2_NaRhBr_6_, and K_2_NaRhI_6_, respectively. The HSE06 functional predicts significantly larger values of 4.829 eV, 3.204 eV, 2.660 eV, and 1.673 eV for the corresponding compounds. The larger band gaps obtained from HSE06 originate from the incorporation of a fraction of exact Hartree–Fock exchange, which effectively reduces the self-interaction error commonly associated with conventional GGA functionals and provides a more accurate description of the electronic structure.

**Fig. 3:**
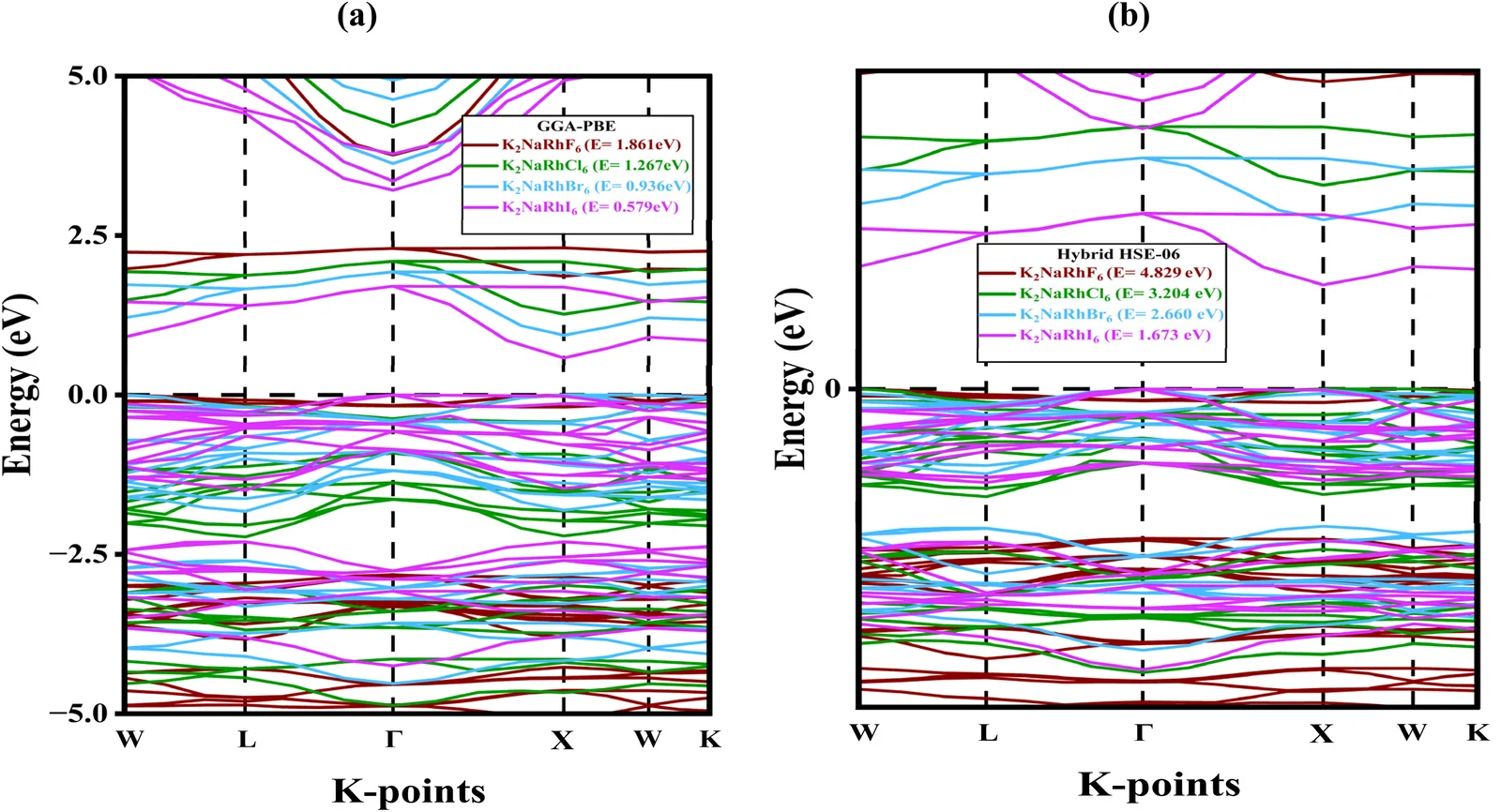
Electronic band structures of K_2_NaRhX_6_ (where X = F, Cl, Br, I) perovskites calculated using (a) GGA-PBE and (b) HSE-06 functionals.

Both computational approaches exhibit the same compositional trend: the band gap decreases systematically from fluoride to iodide. This behavior can be attributed to the increasing ionic radius and decreasing electronegativity of the halogen atoms from F^−^ to I^−^. The larger and more diffuse p orbitals of the heavier halogens enhance orbital overlap with Rh states, resulting in an upward shift of the valence-band edge and a consequent narrowing of the forbidden energy region.^56^ The observed band-gap trend has important implications for device applications. The relatively small band gaps of K_2_NaRhBr_6_ and K_2_NaRhI_6_ indicate strong absorption in the visible region, making them promising candidates for photovoltaic and visible-light optoelectronic devices. In contrast, the wider band gaps of K_2_NaRhF_6_ and K_2_NaRhCl_6_ suggest potential applications in ultraviolet optoelectronics, transparent semiconductor technologies, and high-energy photonic devices. Although the HSE06 functional generally provides more accurate band-gap predictions, it is computationally considerably more demanding than the GGA-PBE approach because of the inclusion of exact exchange interactions.^57^ For large double-perovskite systems, HSE06 calculations require substantially greater computational resources and significantly longer execution times. Therefore, HSE06 was employed primarily to validate the electronic structure and band-gap trends, whereas the subsequent electronic, optical, and transport-property calculations were performed using the computationally efficient GGA-PBE functional. Importantly, both methods predict the same direct-band-gap nature and identical compositional trends, indicating that the qualitative physical conclusions remain unchanged.

#### 3.5.1. Partial and total density of states (PDOS and TDOS) analysis

Fig. 4 shows the total density of states (TDOS) of K_2_NaRhX_6_ (X = F, Cl, Br, I) double perovskites calculated using the GGA-PBE functional. The TDOS is plotted over the energy range from −6 to +6 eV, with the Fermi level fixed at 0 eV. As shown in Fig. 4, all compounds exhibit a clear depletion of states around the Fermi level, confirming their semiconducting nature. The width of the zero-DOS region gradually decreases from K_2_NaRhF_6_ to K_2_NaRhI_6_, indicating a systematic reduction in the band gap with increasing halogen size. This observation is in excellent agreement with the electronic band-structure results.

**Fig. 4:**
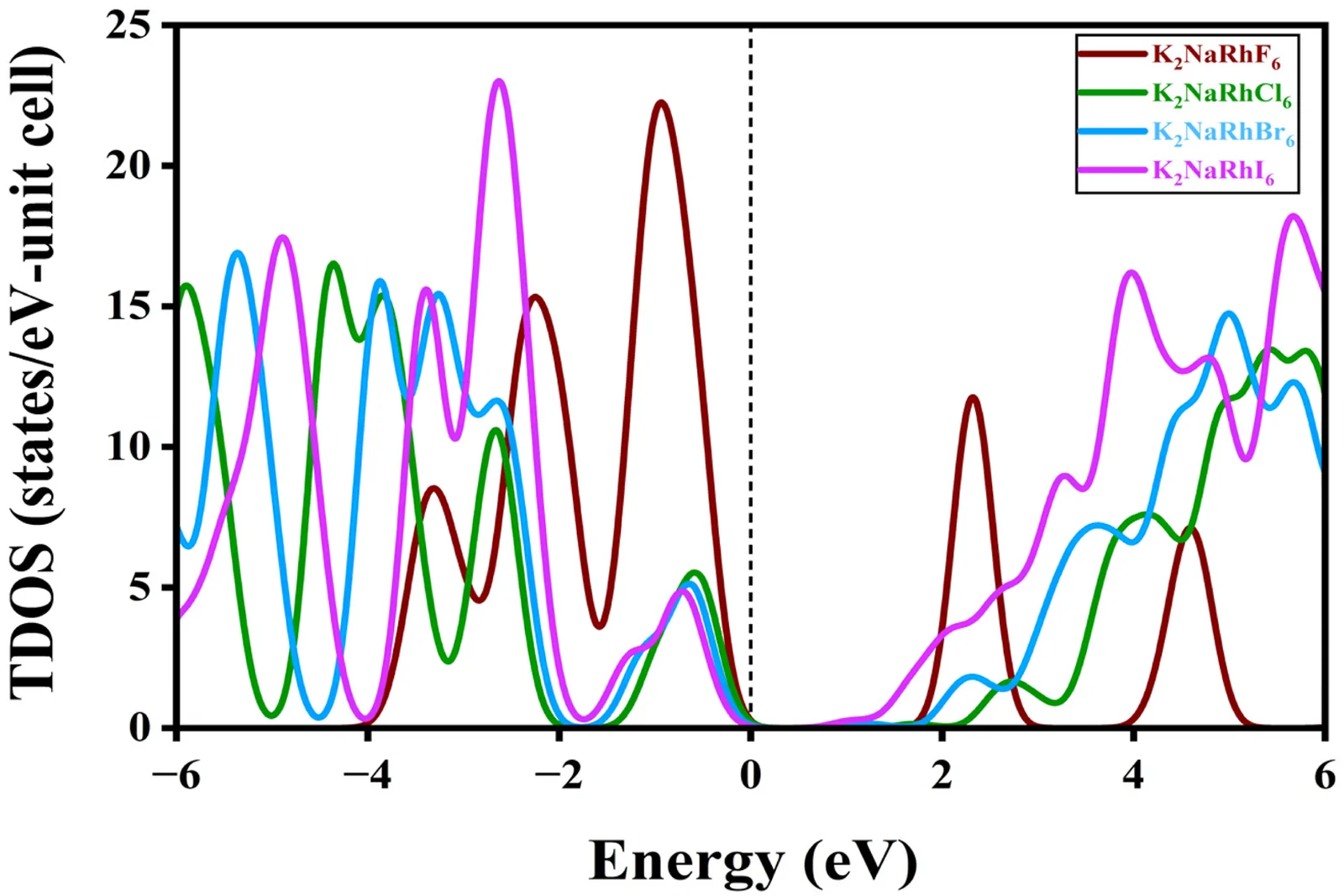
Total density of states of K_2_NaRhX_6_ double perovskite materials.

Furthermore, noticeable shifts in both the valence-band and conduction-band states are observed as the halogen atom changes from F to I. The electronic states progressively move closer to the Fermi level, leading to a narrowing of the forbidden energy region. Among the investigated compounds, K_2_NaRhF_6_ exhibits the widest gap around the Fermi level, whereas K_2_NaRhI_6_ shows the narrowest gap. In addition, the iodide compound displays relatively stronger DOS features near the conduction-band region, suggesting a greater availability of electronic states for carrier excitation. Overall, the TDOS analysis confirms the semiconducting behavior of all K_2_NaRhX_6_ double perovskites and demonstrates that halogen substitution effectively tunes their electronic structure. A more detailed understanding of the orbital contributions to the electronic states is provided in the projected density of states (PDOS) analysis.

The projected density of states (PDOS) provides detailed insight into the atomic and orbital contributions to the electronic structure of K_2_NaRhX_6_ (X = F, Cl, Br, I) double perovskites. The calculated PDOS for the investigated compounds is presented in Fig. 5(a–d), where the Fermi level is set at 0 eV. As shown in Fig. 5, the valence-band region extending from approximately −5 eV to the Fermi level is predominantly composed of halogen p states, namely F-2p, Cl-3p, Br-4p, and I-5p orbitals. A smaller contribution from Rh-4d states is also observed near the valence-band edge, indicating p–d hybridization between the halogen and Rh orbitals. This hybridization plays an important role in determining the electronic structure near the band gap. In contrast, the conduction-band region is mainly dominated by Rh-4d states, demonstrating that the low-energy electronic excitations primarily originate from Rh-centered orbitals. The alkali-metal ions K and Na contribute only weakly near the Fermi level, suggesting that they mainly act as charge-balancing species and help stabilize the crystal framework rather than directly participating in the band-edge electronic transitions. A systematic evolution of the electronic states is observed across the halogen series. From K_2_NaRhF_6_ to K_2_NaRhI_6_, the halogen-p states gradually shift toward the Fermi level, leading to a reduction in the separation between the valence- and conduction-band edges. This trend is fully consistent with the band-structure and TDOS results, which predict a progressive decrease in the band gap from fluoride to iodide.

**Fig. 5:**
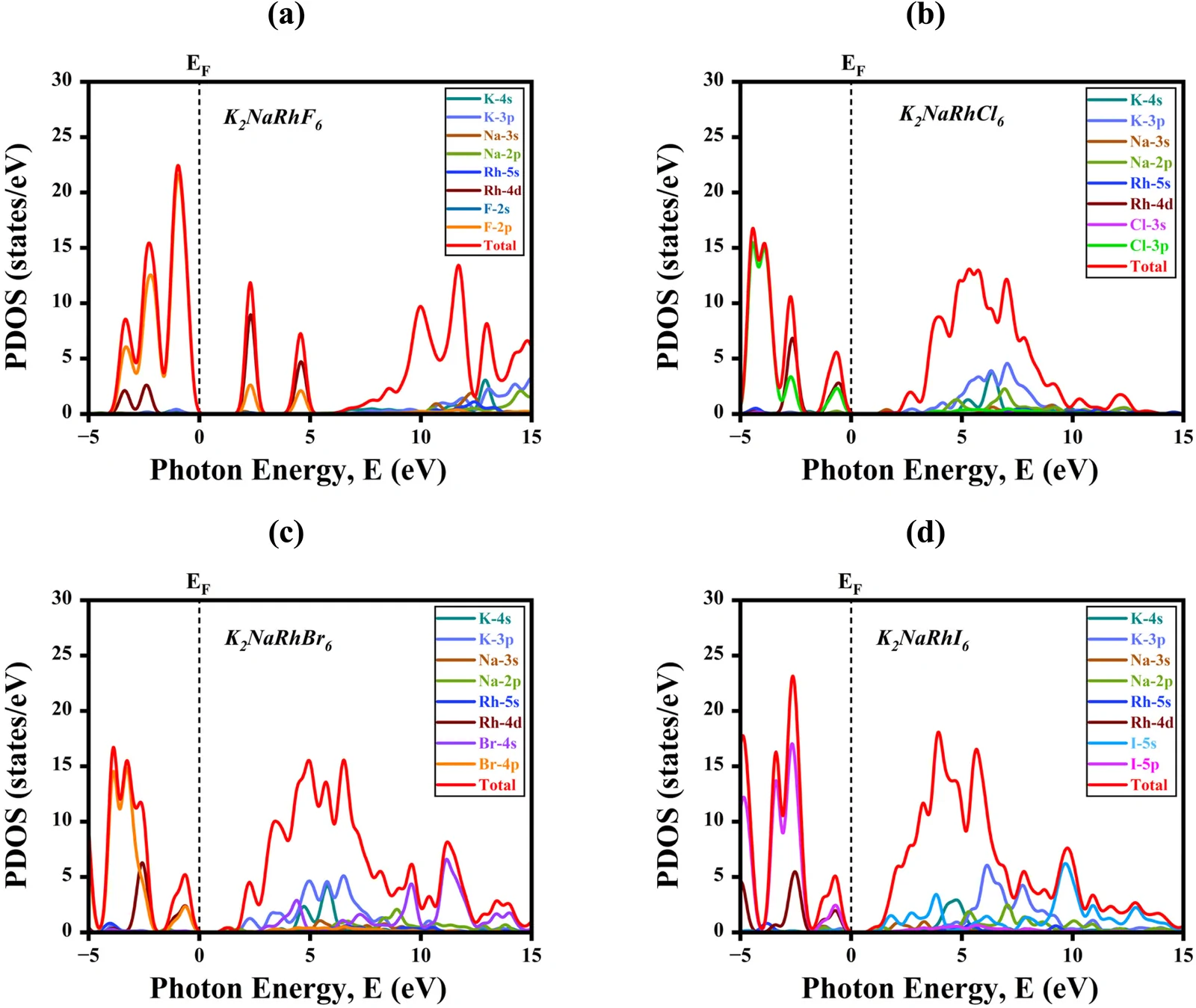
Projected density of states (PDOS) of (a) K_2_NaRhF_6_, (b) K_2_NaRhCl_6_, (c) K_2_NaRhBr_6_, and (d) K_2_NaRhI_6_ calculated using the GGA-PBE functional.

Overall, the PDOS analysis reveals that the electronic properties of K_2_NaRhX_6_ double perovskites are primarily governed by the interaction between Rh-4d and halogen-p orbitals, while K and Na make only minor contributions near the Fermi level. The tunability of the band-edge states through halogen substitution highlights the potential of these materials for optoelectronic and photovoltaic applications.

#### 3.5.2. DFT-derived electronic parameters for device simulation

To establish a reliable connection between the first-principles calculations and photovoltaic device simulations, several electronic parameters were extracted from the DFT results and employed as input parameters in the SCAPS-1D model. The calculated parameters include the band gap (*E*_g_), electron affinity (*χ* (eV)), effective density of states in the conduction band (*N*_C_) and valence band (*N*_V_), carrier effective masses (*m*_e_ and *m*_h_), and carrier mobilities (*µ*_e_ and *µ*_h_). The corresponding values are summarized in Table 3.

**Table 3:** DFT-derived electronic parameters of K_2_NaRhX_6_ (X = F, Cl, Br, I) double perovskites used as input parameters for SCAPS-1D simulations

Parameters	K_2_NaRhF_6_	K_2_NaRhCl_6_	K_2_NaRhBr_6_	K_2_NaRhI_6_
Band gap, *E*_g_ (eV)	1.861	1.267	0.936	0.579
Electron affinity, *χ* (eV)	3.239	3.833	4.164	4.521
*C*_B_ effective density of states, *N*_C_ (cm^−3^)	8.43 × 10^18^	1.007 × 10^19^	1.238 × 10^19^	1.247 × 10^19^
*V*_B_ effective density of states, *N*_V_ (cm^−3^)	1.512 × 10^19^	1.515 × 10^19^	1.515 × 10^19^	1.503 × 10^19^
Electron effective mass, 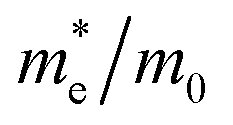	1.125	0.9275	0.834	0.753
Hole effective mass, 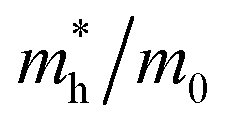	1.572	1.3965	1.345	1.225
Electron mobility, *µ*_n_ (cm^2^ V^−1^ s^−1^)	46.93	56.94	63.31	70.11
Hole mobility, *µ*_h_ (cm^2^ V^−1^ s^−1^)	33.61	37.79	39.27	43.12

The band-gap and electron-affinity values were obtained directly from the electronic-structure calculations. The carrier effective masses (*m*_e_ and *m*_h_) were determined from the curvature of the conduction-band minimum (CBM) and valence-band maximum (VBM), respectively, using the parabolic approximation near the band extrema. The effective densities of states (*N*_C_ and *N*_V_) were subsequently estimated from the calculated carrier effective masses within the parabolic-band approximation. The carrier mobilities were evaluated using the relaxation-time approximation according to 58,4
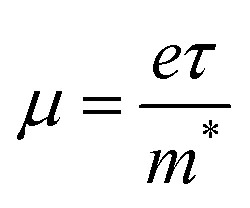
where *e* is the elementary charge, *τ* is the carrier relaxation time, and m* is the carrier effective mass. A relaxation time of 3 × 10^−14^ s was assumed for all compounds within the constant relaxation time approximation. Since explicit electron–phonon scattering calculations were not performed, this value was adopted from the literature and was used to enable a consistent comparative evaluation of carrier mobility among the K_2_NaRhX_6_ compounds. As shown in Table 3, a clear compositional trend is observed across the K_2_NaRhX_6_ series. The band gap decreases systematically from 1.861 eV for K_2_NaRhF_6_ to 0.579 eV for K_2_NaRhI_6_, while the electron affinity increases from 3.239 eV to 4.521 eV. Simultaneously, both electron and hole effective masses decrease with increasing halogen size, resulting in enhanced carrier mobilities. The electron mobility increases from 46.93 cm^2^ V^−1^ s^−1^ for K_2_NaRhF_6_ to 70.11 cm^2^ V^−1^ s^−1^ for K_2_NaRhI_6_, whereas the hole mobility increases from 33.61 to 43.12 cm^2^ V^−1^ s^−1^. Furthermore, the conduction-band effective density of states exhibits a gradual increase across the series, while the valence-band effective density of states remains nearly unchanged. The reduction in carrier effective masses and the corresponding enhancement of carrier mobility indicate improved charge-transport characteristics for the heavier halide compounds, particularly K_2_NaRhBr_6_ and K_2_NaRhI_6_. Therefore, these DFT-derived electronic parameters provide a physically consistent and reliable basis for the subsequent SCAPS-1D photovoltaic simulations.

### 3.6. Optical properties

The optical response of K_2_NaRhX_6_ was evaluated from the complex dielectric function, from which the absorption coefficient, optical conductivity, reflectivity, refractive index and energy-loss function were derived; the corresponding expressions are given with each subsection.

#### 3.6.1. Dielectric function, *ε*(*ω*)

The complex dielectric function (*ε*) describes how a material responds to the oscillating electric field of light. The real part describes the dispersive (polarization) response of the material and the imaginary part the absorptive response. Eqn (9) and (10) are the expressions for the dielectric function.^59^9
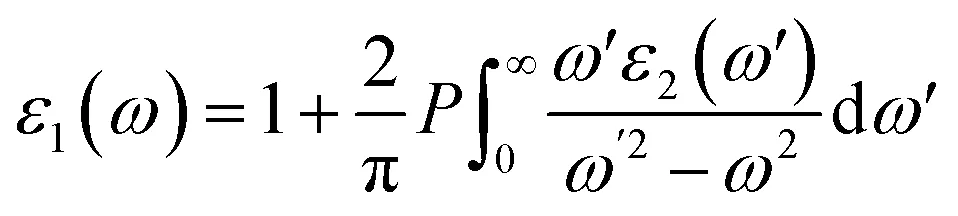
10
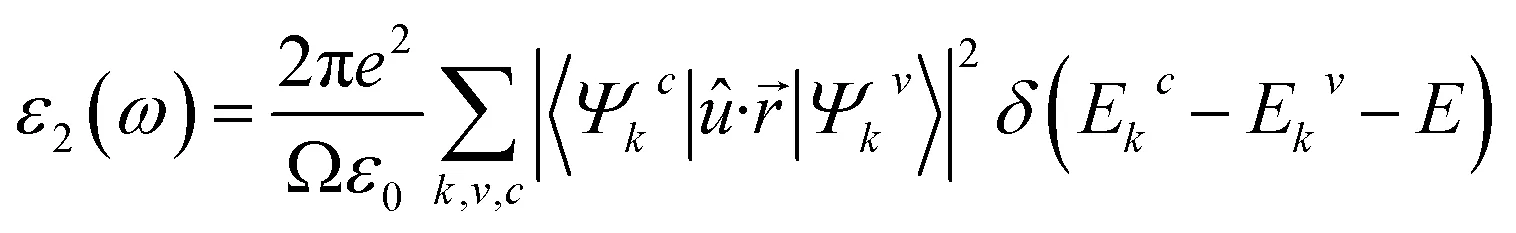


Fig. 6(a) illustrates the real (*ε*_1_) and imaginary (*ε*_2_) parts of the dielectric function as a function of photon energy. At 0 eV, *ε*_1_ values are 2.353, 3.696, 4.579, and 6.428 for K_2_NaRhF_6_, K_2_NaRhCl_6_, K_2_NaRhBr_6_, and K_2_NaRhI_6_, respectively. These static dielectric constants were subsequently employed as the relative dielectric permittivity (*ε*_r_) in the SCAPS-1D device simulations. As photon energy increases, *ε*_1_ rises and exhibits several prominent peaks associated with interband electronic transitions. K_2_NaRhI_6_ shows the highest *ε*_1_ value of 7.0 at 1.1 eV, followed by K_2_NaRhBr_6_ (5.5 at 1.8 eV) and K_2_NaRhCl_6_ (4.6 at 2.7 eV), indicating enhanced electronic polarization and dielectric screening. At higher photon energies, *ε*_1_ gradually decreases, and zero-crossing behavior is observed for K_2_NaRhI_6_, K_2_NaRhBr_6_, and K_2_NaRhCl_6_. The most pronounced crossing occurs for K_2_NaRhI_6_ at 8.8 eV, where *ε*_1_ becomes negative before returning to positive values near 11 eV. Such behavior is characteristic of plasma-like responses and enhanced reflectivity in the high-energy region. The imaginary part, *ε*_2_, which represents optical absorption arising from interband transitions, exhibits absorption onsets at approximately 0, 0.9, 1.0, and 4.0 eV for K_2_NaRhI_6_, K_2_NaRhBr_6_, K_2_NaRhCl_6_, and K_2_NaRhF_6_, respectively. As photon energy increases, *ε*_2_ rises steadily and reaches its maximum value of 4.9 at 6.4 eV for K_2_NaRhI_6_. The remaining compounds also display prominent *ε*_2_ peaks in the UV region, reflecting strong optical absorption. Unlike *ε*_1_, the imaginary component does not exhibit zero-crossing behavior throughout the investigated energy range.

**Fig. 6:**
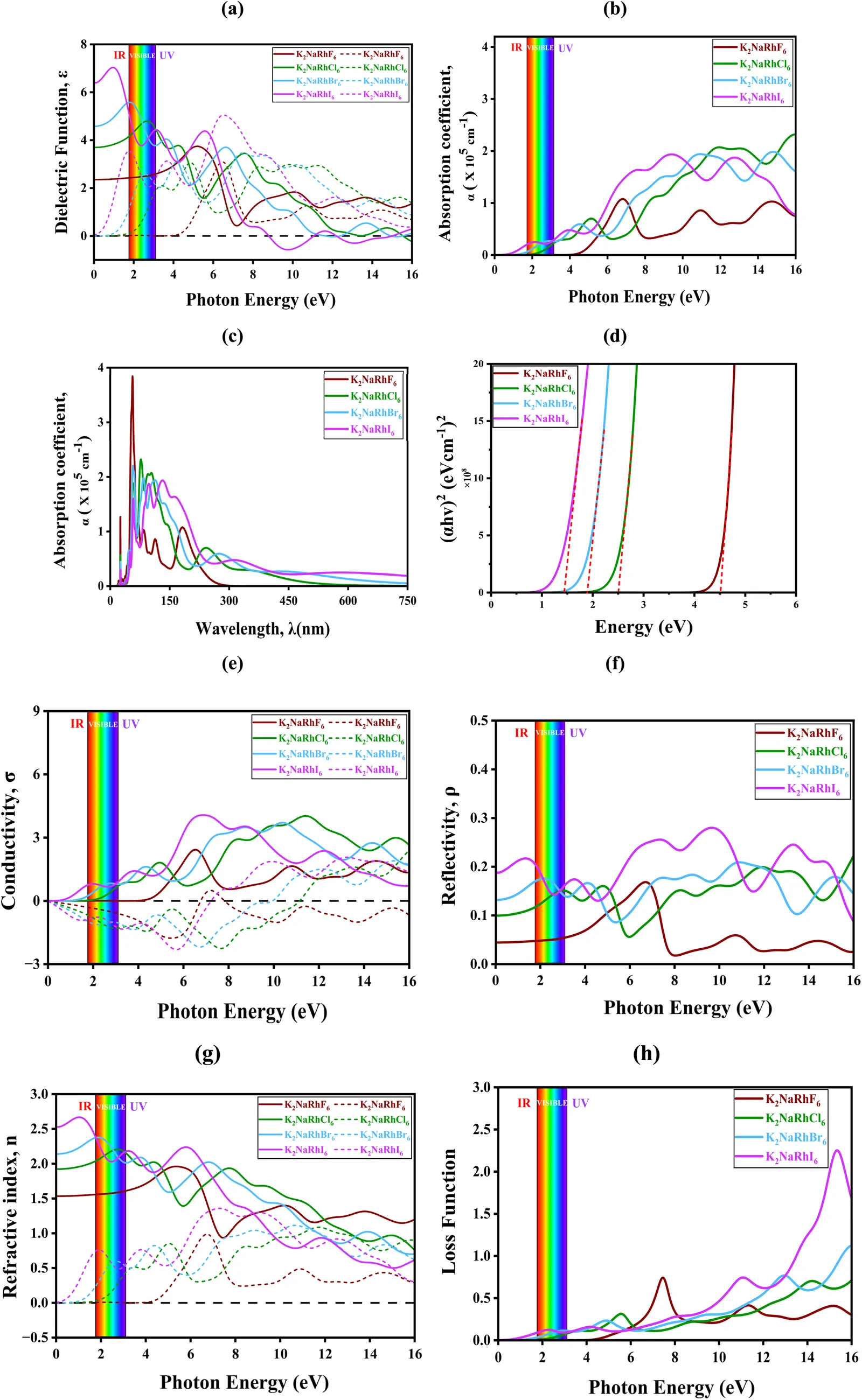
Calculated optical functions of K_2_NaRhF_6_ (where X = F, Cl, Br, I) perovskites: (a) dielectric function, (b and c) absorption coefficient, (d) optical band gap from absorption spectra *via* Tauc plot (e) optical conductivity, (f) reflectivity, (g) refractive index, and (h) energy loss function.

#### 3.6.2. Absorption coefficient (*α*)

A high *α* in the visible region is desirable for photovoltaic applications, as it enables efficient light harvesting in thin absorber layers. In DFT, *α* is derived from the complex dielectric function, where *ε*_1_(*ω*) and *ε*_2_(*ω*) are the real and imaginary parts, respectively.5
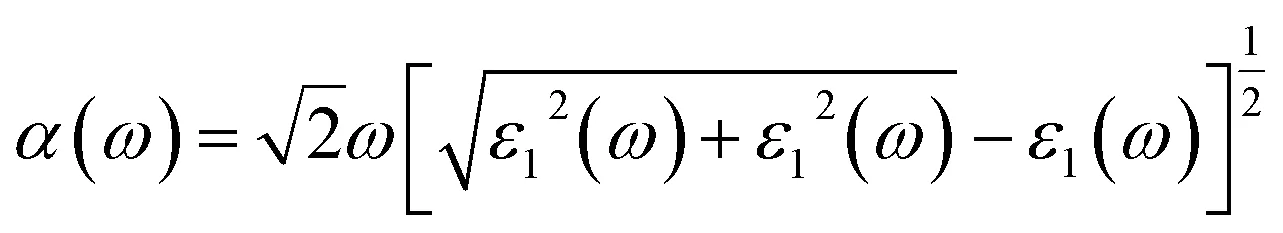


Fig. 6(b) presents the absorption coefficient as a function of photon energy. The absorption onset shifts toward lower energies from F to I, reflecting the reduction in optical bandgap. K_2_NaRhI_6_, K_2_NaRhBr_6_, and K_2_NaRhCl_6_ begin absorbing at approximately 0.9, 1.2, and 1.9 eV, respectively, whereas K_2_NaRhF_6_ exhibits absorption onset near 4.0 eV. The maximum absorption coefficients are observed in the UV region, reaching 2.4 × 10^5^ cm^−1^ at 16 eV for K_2_NaRhCl_6_, 2.2 × 10^5^ cm^−1^ at 14.7 eV for K_2_NaRhBr_6_, and 2.0 × 10^5^ cm^−1^ at 9.1 eV for K_2_NaRhI_6_, indicating strong high-energy photon absorption. Fig. 6(c) depicts the wavelength-dependent absorption spectra. Strong absorption is concentrated in the UV region below 150 nm, where K_2_NaRhF_6_ exhibits the highest absorption coefficient of 3.9 × 10^5^ cm^−1^ at 60 nm, followed by K_2_NaRhCl_6_ with 2.3 × 10^5^ cm^−1^ at 75 nm. K_2_NaRhBr_6_ and K_2_NaRhI_6_ show peak absorption coefficients of 2.2 × 10^5^ cm^−1^ at 58 nm and 1.9 × 10^5^ cm^−1^ at 140 nm, respectively. Notably, K_2_NaRhBr_6_ and K_2_NaRhI_6_ extend furthest into the visible and near-infrared regions, suggesting enhanced utilization of lower-energy photons. The optical band gap was estimated from the calculated absorption coefficient using the Tauc relation. The optical band gaps extracted from the Tauc plots in Fig. 6(d) are 4.503, 2.506, 1.899, and 1.449 eV for K_2_NaRhF_6_, K_2_NaRhCl_6_, K_2_NaRhBr_6_, and K_2_NaRhI_6_, respectively. The bandgap decreases systematically in the order F > Cl > Br > I with increasing halogen atomic size, resulting in improved sensitivity to lower-energy photons. Consequently, K_2_NaRhI_6_ emerges as the most promising candidate for photovoltaic applications, whereas K_2_NaRhF_6_ is more suitable for UV-optoelectronic devices.

#### 3.6.3. Conductivity (*σ*)

Optical conductivity is a calculated optical property that describes a material's electronic response to an oscillating electromagnetic field and is derived from the complex dielectric function.^60,61^Fig. 6(e), the solid lines represent the real part of conductivity, and the dashed lines represent the imaginary part. No noticeable response appears in the infrared (IR) region for any compound. The real part of F-based compounds shows no peak at all up to a certain energy source in the UV region. However, K_2_NaRhCl_6_, K_2_NaRhBr_6_, and K_2_NaRhI_6_ start to show moderate peaks throughout the IR-VR transition region and in the VR region. With further increase in photon energy into the UV region, the real part of the conductivity increases for all compounds and corresponds to multiple distinct peaks across the energy spectrum. In the UV region, they exhibit high conductivity. 2 at 6.7 eV for K_2_NaRhF_6_, 3.9 at 11.8 eV for K_2_NaRhCl_6_, 3.5 at 10.4 for K_2_NaRhBr_6_ and 4 at 7 eV for K_2_NaRhI_6_. This demonstrates strong optical conductivity. The imaginary part of the conductivity is also shown in Fig. 6(e). At low photon energies, negative values are exhibited initially. As photon energy is increased, maximum negative peaks are reached in the UV region, which are −1.8 at 5.7 eV for K_2_NaRhF_6_, −2.3 at 7.7 eV for K_2_NaRhCl_6_, −2.2 at 6.6 eV for K_2_NaRhBr_6_ and −2.4 at 5.8 eV for K_2_NaRhI_6_. Zero-crossing behavior is observed for all four compounds. K_2_NaRhCl_6_ crosses at 11 eV, K_2_NaRhBr_6_ crosses at 9.8 eV and K_2_NaRhI_6_ crosses at 7.2 eV. Their imaginary part of conductivity shows a positive result after going upward. However, K_2_NaRhF_6_ crosses zero at 6.7 eV and shows very little positive value and afterwards crosses zero again remain in the negative zone. The observed zero-crossing of the imaginary part reflects the energy-dependent evolution of the optical response and is associated with changes in interband electronic transitions.

#### 3.6.4. Reflectivity (*ρ*)

Reflectivity (*R*) is the ratio of reflected electromagnetic radiation to the total incident light intensity.^62,63^ In DFT, *R* is calculated from the complex dielectric function using the following expression:6
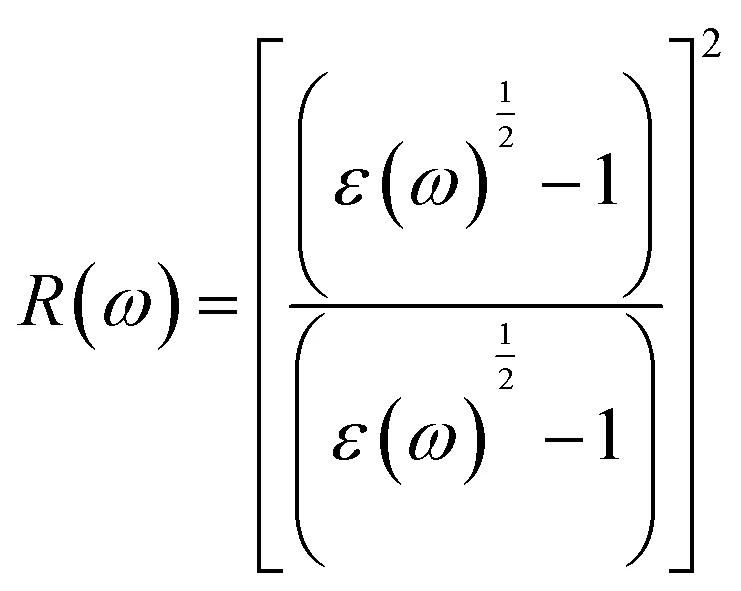


Fig. 6(f) illustrates the reflectivity spectra of K_2_NaRhX_6_ compounds. At low photon energies, the reflectivity ranges from 0.045 to 0.18, indicating weak reflection in the IR region. As the photon energy increases, K_2_NaRhI_6_, K_2_NaRhBr_6_, and K_2_NaRhCl_6_ exhibit moderate reflectivity in the near-IR region, while pronounced peaks emerge in the UV range. The highest reflectivity of 0.28 is observed for K_2_NaRhI_6_ at 9.9 eV, followed by 0.24 for K_2_NaRhCl_6_ at 16 eV. In contrast, K_2_NaRhF_6_ exhibits the lowest reflectivity throughout the studied energy range, remaining below 0.15. These results indicate that the optical response is strongly influenced by halogen substitution, with I- and Cl-based compounds showing enhanced reflectivity at higher photon energies. The relatively high reflectivity of K_2_NaRhI_6_ and K_2_NaRhCl_6_ suggests potential applicability in UV-visible optoelectronic devices and protective coatings, whereas the low reflectivity of K_2_NaRhF_6_ favors efficient photon absorption, making it attractive for photovoltaic and light-harvesting applications.

#### 3.6.5. Refractive index, (*ω*)

The refractive index (*n*) is a constant that describes how light propagates through a medium compared to a vacuum. In CASTEP simulations, *n*(*ω*) and *k*(*ω*) are computed from the complex dielectric function, using the relation,^64^7
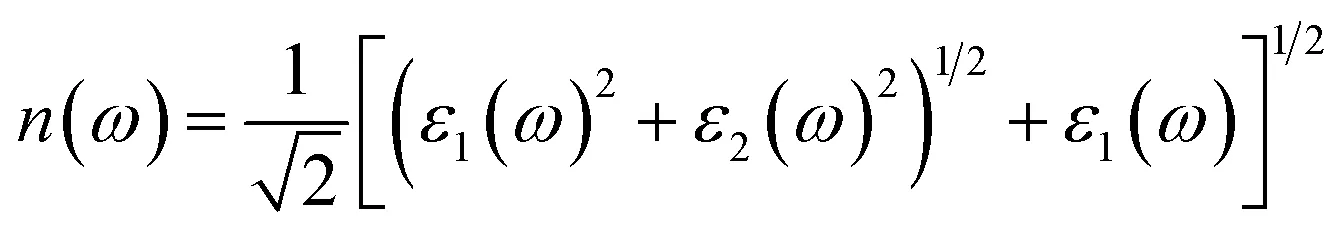
8
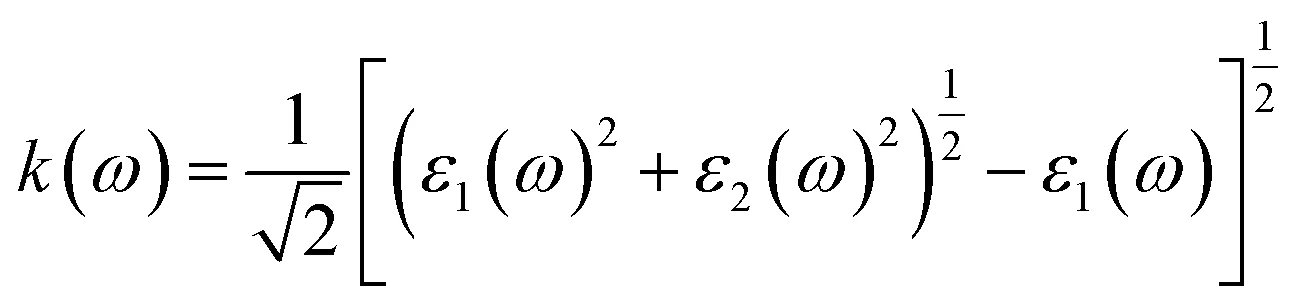


Fig. 6(g) shows the photon-energy dependence of the refractive index, where solid and dashed lines represent the real (*n*) and imaginary (*k*) components, respectively. At 0 eV, the static refractive indices are approximately 1.55, 1.9, 2.2, and 2.5 for K_2_NaRhF_6_, K_2_NaRhCl_6_, K_2_NaRhBr_6_, and K_2_NaRhI_6_, respectively. The real part generally increases with photon energy and reaches maximum values of 1.95, 2.2, 2.4, and 2.6 for the F-, Cl-, Br-, and I-based compounds, respectively. The highest peak is observed for K_2_NaRhI_6_ (2.6 at 1.2 eV), followed by K_2_NaRhBr_6_ (2.4 at 1.9 eV). Beyond these energies, *n* gradually decreases, although K_2_NaRhCl_6_, K_2_NaRhBr_6_, and K_2_NaRhI_6_ exhibit a slight rise above 4 eV. In contrast, K_2_NaRhF_6_ decreases markedly from 1.9 to 1.0 between 5 and 7.8 eV. Overall, the refractive index follows the trend K_2_NaRhI_6_ > K_2_NaRhBr_6_ > K_2_NaRhCl_6_ > K_2_NaRhF_6_. The imaginary component remains nearly zero in the low-energy region, indicating negligible optical absorption. As photon energy increases, *k* rises progressively, with more pronounced features for K_2_NaRhBr_6_ and K_2_NaRhI_6_ due to their narrower band gaps. In the UV region, all compounds exhibit multiple distinct peaks associated with strong interband electronic transitions. The consistently larger *n* and *k* values of K_2_NaRhI_6_ demonstrate stronger light–matter interaction, highlighting its potential for optoelectronic and photovoltaic applications.

#### 3.6.6. Loss function (*L*(*ω*))

The energy-loss function quantifies the energy dissipated by fast-moving electrons and is defined as,^65^11
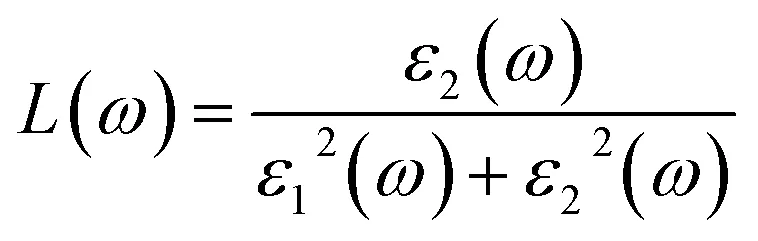


Fig. 6(h) presents the energy-loss function spectra of K_2_NaRhX_6_ compounds. In the IR and visible regions, all materials exhibit negligible energy-loss responses. The highest loss-function peak of 2.3 is observed for K_2_NaRhI_6_ at approximately 15 eV, while K_2_NaRhBr_6_ exhibits the second-highest peak of 1.1 at 16 eV, indicating stronger plasmonic resonance and electron–photon interactions at high photon energies. In contrast, K_2_NaRhCl_6_ shows the lowest loss-function response, suggesting comparatively weaker plasmonic activity. The magnitude of the loss peaks generally increases with halogen size from Cl to I, consistent with enhanced electronic polarizability. The pronounced peak observed for K_2_NaRhI_6_ implies stronger collective electronic excitations, whereas the lower loss response of K_2_NaRhCl_6_ indicates reduced energy dissipation. These results highlight the tunable plasmonic and optical characteristics of K_2_NaRhX_6_ compounds, which may be advantageous for ultraviolet optoelectronic and photonic applications.

### 3.7. Charge density analysis

For K_2_NaRhX_6_ compounds, charge density analysis was employed to examine the electronic distribution around the Rh and halogen sites and to assess the qualitative character of Rh–X interactions. In the charge density maps presented in Fig. 7, the red and yellow regions indicate relatively high electron density, whereas the blue regions correspond to lower electron density. The electron density is mainly concentrated around the halogen and Rh sites, indicating significant charge localization in these regions. The comparatively lower charge localization around Na is consistent with its predominantly ionic contribution to the crystal framework. Across the K_2_NaRhX_6_ series, K_2_NaRhF_6_ exhibits the most localized charge distribution around the Rh–F bonding region, consistent with the high electronegativity and smaller ionic radius of F. In contrast, K_2_NaRhBr_6_ and K_2_NaRhI_6_ show more diffuse charge distributions around the bonding regions because of the larger spatial extent of the heavier halogen orbitals.

**Fig. 7:**
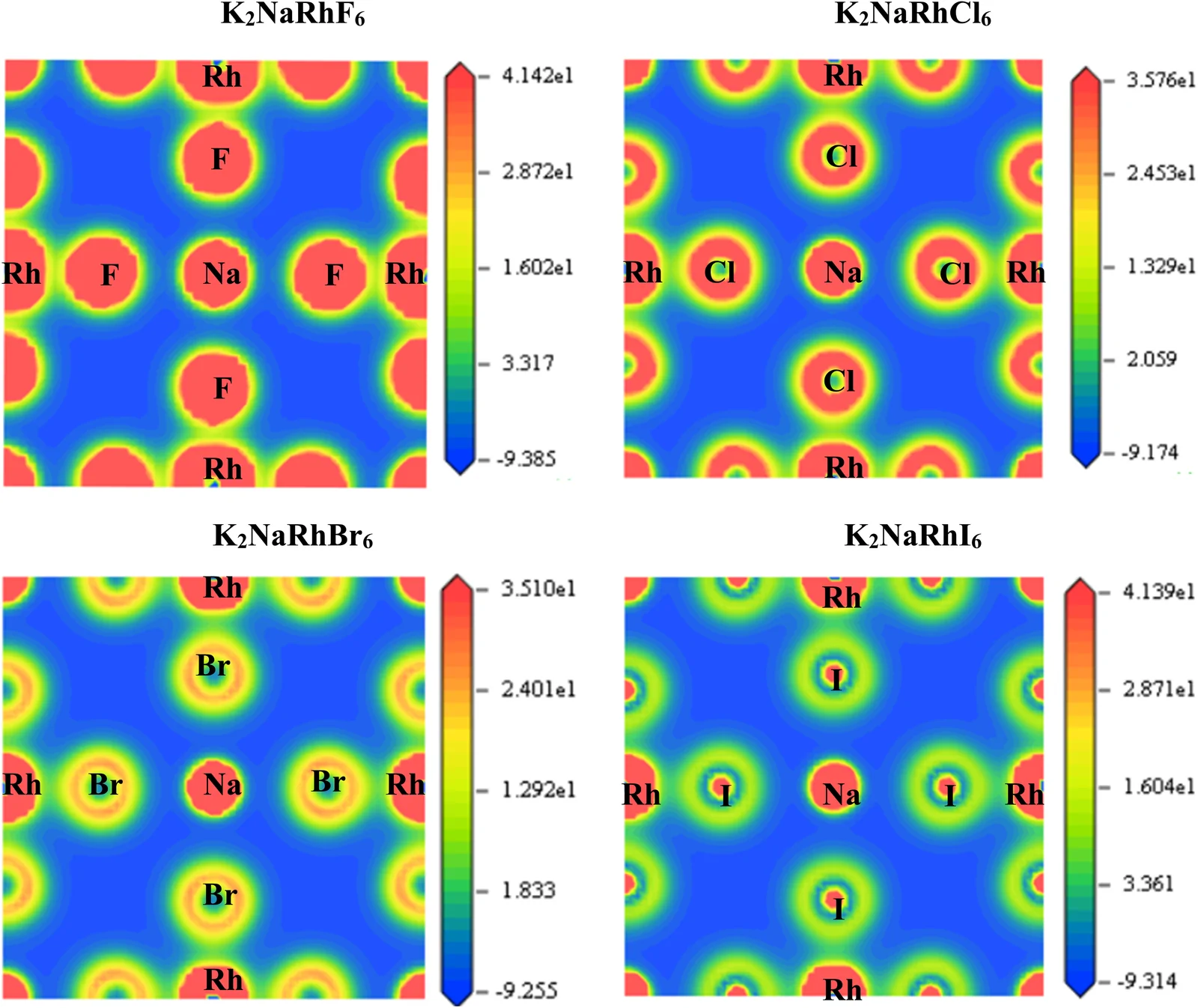
Charge density mapping of K_2_NaRhX_6_ double perovskite materials.

The increase in halogen ionic radius from F to I is accompanied by an increase in lattice parameter from 6.111 to 8.340 Å, which increases the Rh–X bond distances and may reduce orbital overlap between Rh-d and halogen-p states. Consequently, the charge density becomes progressively more diffuse from F to I. These results indicate a gradual variation in charge localization and Rh–X bonding interactions across the halogen series.

### 3.8. Phonon dispersion

Phonon dispersion analysis provides a direct approach for evaluating the dynamical stability of a crystal structure by describing its vibrational modes throughout the Brillouin zone.^66^ The calculated phonon dispersion spectra of K_2_NaRhX_6_ (X = F, Cl, Br, and I) along the selected high-symmetry directions are presented in Fig. S1(a–d) of the SI. For K_2_NaRhF_6_ (Fig. S1(a)), the phonon branches extend up to approximately 8 THz, with most modes exhibiting positive frequencies. A small imaginary-frequency region appears near the *M* point in the low-frequency acoustic branch, indicating a possible weak lattice instability. K_2_NaRhCl_6_ (Fig. S1(b)) exhibits phonon branches extending to approximately 10 THz, with low-frequency soft modes and a small imaginary-frequency region around the *M* point. For K_2_NaRhBr_6_ (Fig. S1(c)), the upper phonon frequency decreases to approximately 7.5 THz, and more noticeable low-frequency imaginary modes are observed near the *M* point. K_2_NaRhI_6_ (Fig. S1(d)) exhibits the lowest upper phonon frequency, approximately 6.1 THz, together with low-frequency imaginary modes. Overall, the lower upper-frequency limit observed for the Br- and I-based compounds is consistent with the increasing halide mass and weakening of Rh–X bonding interactions, indicating progressive lattice softening toward the heavier halides. The low-frequency imaginary modes are mainly concentrated near the Brillouin-zone boundaries, particularly around the *M* point, and may be associated with a tendency toward octahedral tilting or local structural distortions involving the corner-sharing RhX_6_ and NaX_6_ units. Thus, the cubic aristotype may represent an average high-symmetry structure with possible local octahedral distortions.^67^ The qualitative trends in the electronic and optical properties are expected to remain primarily governed by the local RhX_6_ coordination rather than by long-range tilt ordering. It should also be noted that the acoustic branches shown in Fig. S1 do not reach exactly zero frequency at the *Γ* point. This behavior indicates that the low-frequency region is not fully converged. Denser *q*-point sampling, larger supercells, and a higher plane-wave cut-off would be required to establish the low-frequency lattice dynamics quantitatively and to determine whether the observed soft modes persist upon further convergence. Given the computational cost of such calculations, the present phonon results are therefore interpreted as a qualitative indication of lattice softening and possible structural distortion rather than as definitive evidence of dynamical stability. A fully converged treatment of the lattice dynamics using larger supercells and denser sampling is identified as an important direction for future work.

### 3.9. Mechanical properties

The mechanical stability of cubic K_2_NaRhX_6_ compounds can be evaluated from the independent elastic constants (*C*_11_, *C*_12_, and *C*_44_). For a mechanically stable cubic crystal, the Born stability criteria must satisfy:^68–70^

12 (*C*_11_ − *C*_12_ > 0, *C*_11_ + 2*C*_12_ > 0, *C*_44_ > 0)

As shown in Fig. 8, K_2_NaRhF_6_ exhibits the highest (*C*_11_ of 52.85 GPa) and (*C*_44_ of 13.38 GPa), indicating the greatest lattice rigidity, whereas K_2_NaRhI_6_ shows the lowest values of (*C*_11_ of 17.35 GPa) and (*C*_44_ of 3.08 GPa), reflecting a softer and more compressible structure.

**Fig. 8:**
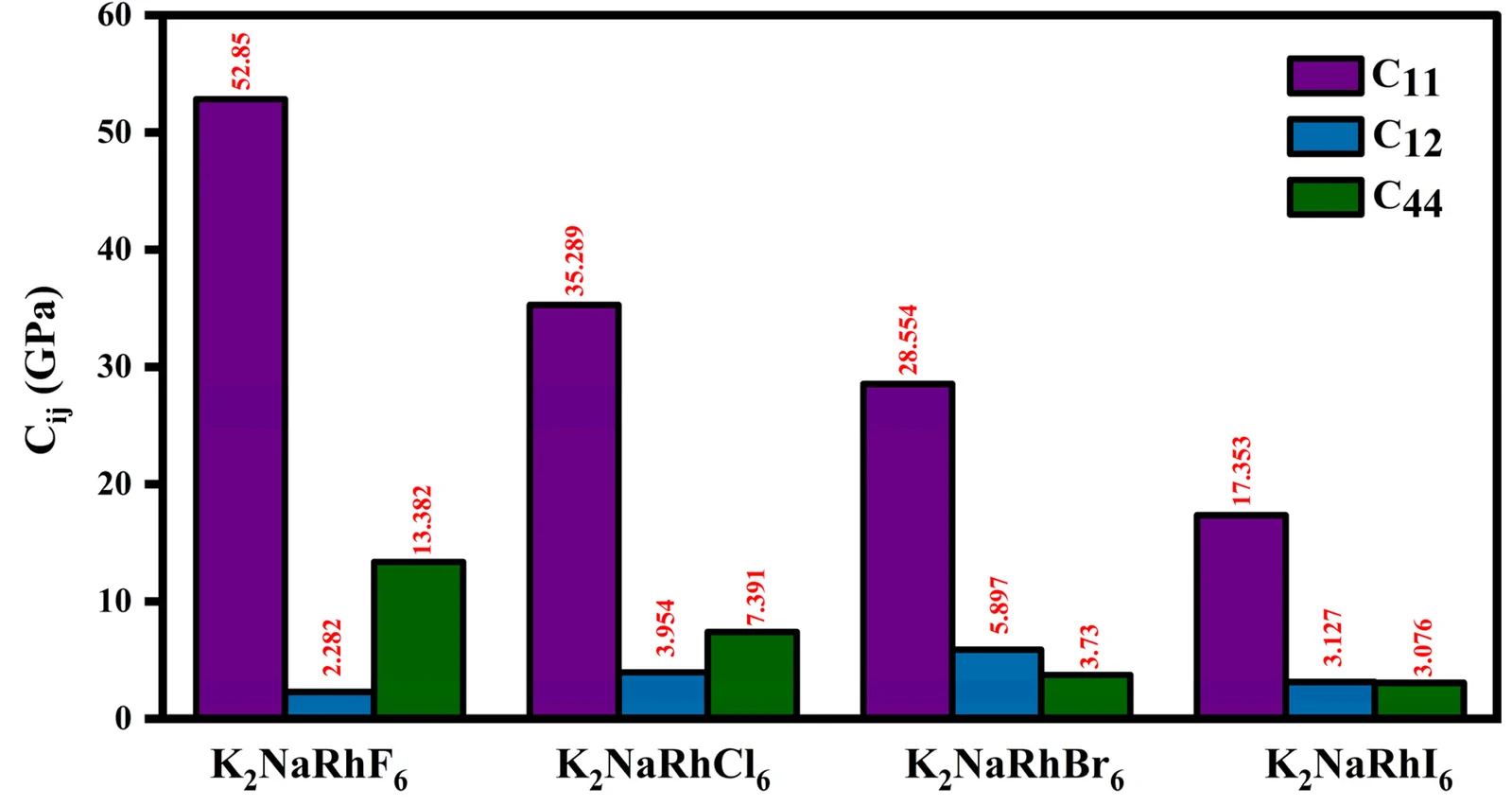
Calculated elastic constants of K_2_NaRhX_6_ (X = F, Cl, Br, I).

The elastic constants decrease systematically in the order K_2_NaRhF_6_ > K_2_NaRhCl_6_ > K_2_NaRhBr_6_ > K_2_NaRhI_6_, which can be attributed to the increasing halogen ionic size and the consequent weakening of Rh–X bonding. Moreover, all compounds satisfy the Born stability criteria, confirming their mechanical stability. These results indicate that halogen substitution effectively tunes the mechanical rigidity of K_2_NaRhX_6_ compounds, ranging from the relatively stiff K_2_NaRhF_6_ to the more compliant K_2_NaRhI_6_.

The bulk modulus (*B*) represents the resistance of a material to uniform volumetric compression and is a measure of incompressibility. It can be calculated for cubic crystals as:^71^13
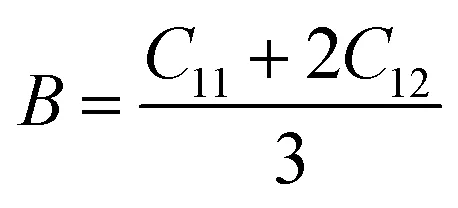


Fig. 9(a) compares the bulk modulus (*B*) values of the cubic double perovskites K_2_NaRhX_6_ (X = F, Cl, Br, I). The calculated bulk modulus values are 19.138 GPa for K_2_NaRhF_6_, 14.399 GPa for K_2_NaRhCl_6_, 13.45 GPa for K_2_NaRhBr_6_, and 7.869 GPa for K_2_NaRhI_6_. A gradual decrease in bulk modules is observed from fluorine to iodine, indicating a reduction in mechanical stiffness with increasing halide ionic size. Since all values are below 40 GPa, the materials are categorized as flexible materials, whereas materials with *B* > 40 GPa are considered hard materials, as indicated by the dashed reference line.

**Fig. 9:**
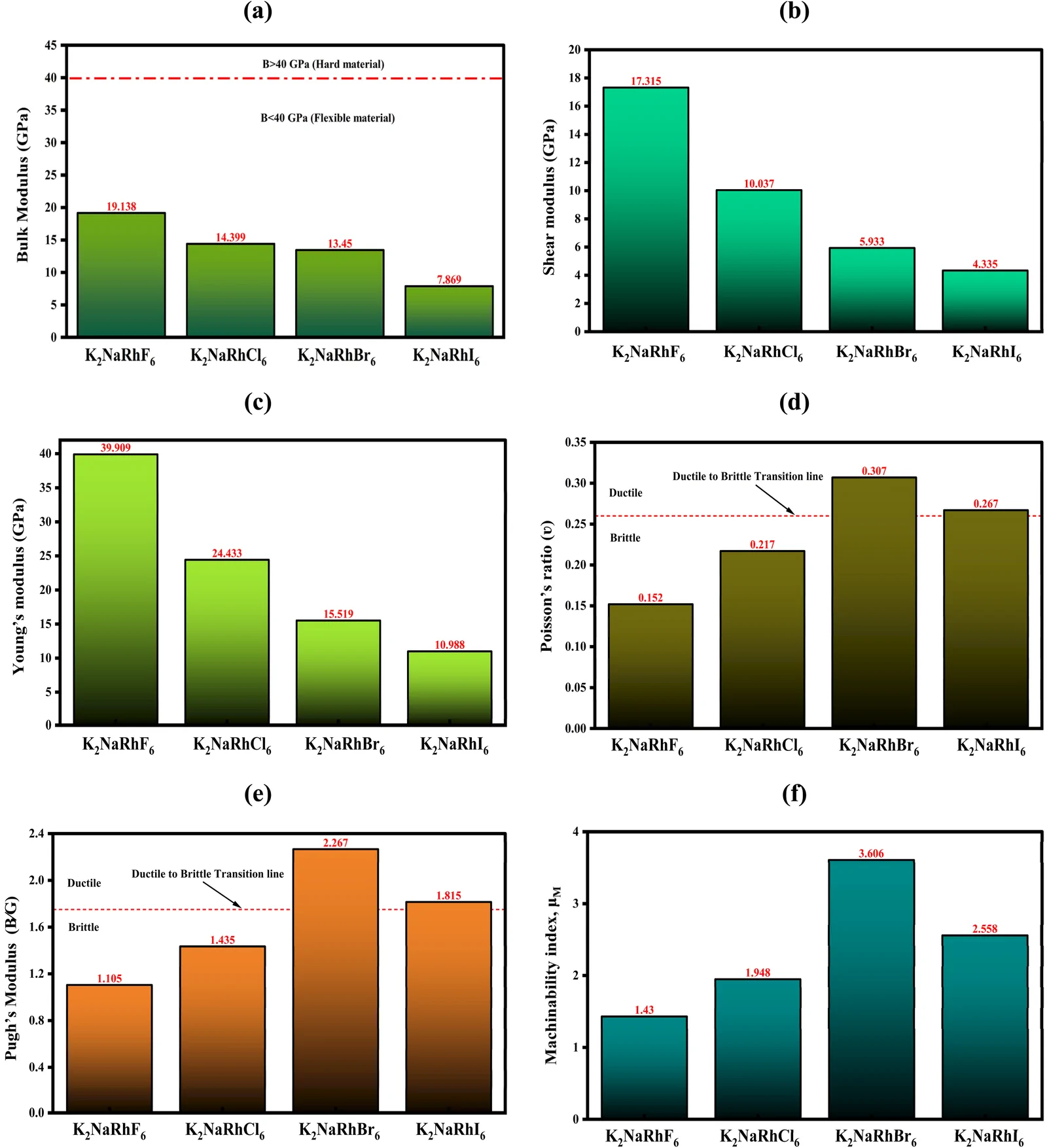
Comparative mechanical properties of K_2_NaRhX_6_ (X = F, Cl, Br, I) double perovskites showing (a) bulk modulus, (b) shear modulus, (c) Young's modulus, (d) Poisson's ratio, (e) Pugh's ratio, and (f) machinability index.

According to the Voigt–Reuss–Hill approximation, the shear modulus is calculated as:14
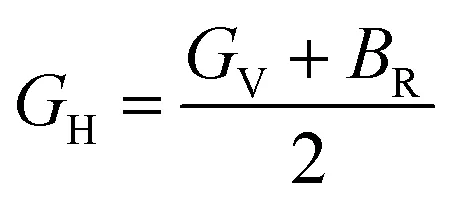


Fig. 9(b) illustrates the variation of shear modulus for the K_2_NaRhX_6_ compounds. The calculated values are 17.315, 10.037, 5.933, and 4.335 GPa for K_2_NaRhF_6_, K_2_NaRhCl_6_, K_2_NaRhBr_6_, and K_2_NaRhI_6_, respectively. Similar to the bulk modulus, the shear modulus decreases systematically from F to I, indicating reduced resistance to shear deformation. K_2_NaRhF_6_ exhibits the highest rigidity, whereas K_2_NaRhI_6_ shows the softest mechanical behavior.

Young's modulus *Y* is calculated using:^72^15
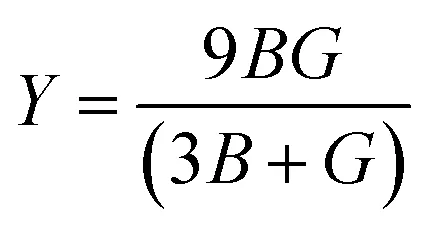


Fig. 9(c) presents the Young's modulus values of K_2_NaRhX_6_. The calculated values are 39.909, 24.433, 15.519, and 10.988 GPa for K_2_NaRhF_6_, K_2_NaRhCl_6_, K_2_NaRhBr_6_, and K_2_NaRhI_6_, respectively. The decreasing trend from fluorine to iodine indicates a gradual reduction in elastic stiffness. Consequently, K_2_NaRhF_6_ exhibits the highest rigidity, whereas K_2_NaRhI_6_ possesses the lowest resistance to elastic deformation.

Poisson's ratio (*ν*) and Pugh's ratio (*B*/*G*) are commonly used to determine the ductile or brittle nature of materials. They are expressed as:^73^16
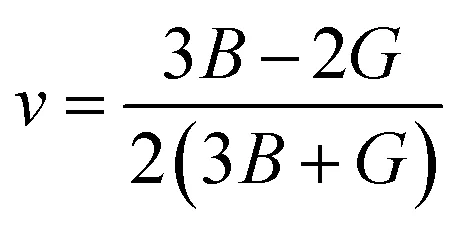


Fig. 9(d) shows the Poisson's ratio values of 0.152, 0.217, 0.307, and 0.267 for K_2_NaRhF_6_, K_2_NaRhCl_6_, K_2_NaRhBr_6_, and K_2_NaRhI_6_, respectively.

Based on the critical value of *ν* = 0.26, K_2_NaRhF_6_ and K_2_NaRhCl_6_ are classified as brittle materials, whereas K_2_NaRhBr_6_ and K_2_NaRhI_6_ exhibit ductile behavior. A similar trend is observed from Fig. 9(e), where the calculated *B*/*G* values are 1.105, 1.435, 2.267, and 1.815, respectively. Since the critical value for ductility is *B*/*G* = 1.75, K_2_NaRhBr_6_ and K_2_NaRhI_6_ are identified as ductile compounds, while K_2_NaRhF_6_ and K_2_NaRhCl_6_ remain brittle.

The machinability index (*µ*_M_) is given by:^74^17
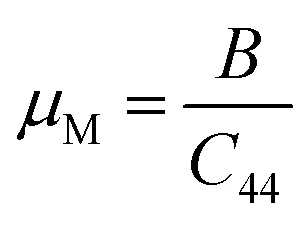


Fig. 9(f) presents the machinability index values of 1.43, 1.95, 3.61, and 2.56 for K_2_NaRhF_6_, K_2_NaRhCl_6_, K_2_NaRhBr_6_, and K_2_NaRhI_6_, respectively. The machinability index increases from fluorine to bromine and then decreases slightly for iodine. Among the investigated compounds, K_2_NaRhBr_6_ exhibits the highest machinability, indicating superior mechanical processability, whereas K_2_NaRhF_6_ shows the lowest machinability due to its higher stiffness and shear resistance.

Overall, the results demonstrate that halogen substitution effectively tunes the rigidity, ductility, and processability of K_2_NaRhX_6_ compounds, with K_2_NaRhBr_6_ and K_2_NaRhI_6_ exhibiting enhanced mechanical flexibility that may be advantageous for thin-film optoelectronic and photovoltaic applications.

### 3.10. Population analysis

Mulliken and Hirshfeld population analyses were employed to examine charge redistribution and the qualitative bonding character of K_2_NaRhX_6_ (X = F, Cl, Br, I) double perovskites.^75,76^ Both approaches indicate that K and Na generally act as electron-donating centers, whereas the halogen atoms exhibit greater electron accumulation. In Table 4, for K_2_NaRhF_6_, the Mulliken charges of K and Na are +0.88 and +0.56, respectively, while F carries a charge of −0.51. The Rh atom has a positive Mulliken charge of +0.71, indicating charge redistribution involving the Rh–F framework. Across the series, the Rh Mulliken charge decreases from +0.71 for K_2_NaRhF_6_ to −0.97 for K_2_NaRhI_6_, while the corresponding halogen charge changes from −0.51 for F to +0.07 for I (Table 4). This variation indicates a systematic change in charge redistribution and bonding character with increasing halogen size and decreasing electronegativity.

**Table 4:** Mulliken and Hirshfeld charge analysis of different atoms of K_2_NaRhX_6_ double perovskites

Compound	Charge spilling	Species	Mulliken atomic populations					Mulliken charge	Hirshfeld charge
			s	p	d	f	Total		
K_2_NaRhF_6_	0.10%	K	2.06	5.96	0.09	0.00	8.12	0.88	0.34
		Na	2.12	6.79	6.33	0.00	8.44	0.56	0.25
		Rh	2.39	6.56	7.34	0.00	16.29	0.71	0.41
		F	1.95	5.56	0.00	0.00	7.51	−0.51	−0.23
K_2_NaRhCl_6_	0.12%	K	2.05	6.02	0.11	0.00	8.18	0.82	0.24
		Na	2.16	6.42	0.00	0.00	8.58	0.42	0.20
		Rh	2.51	6.75	7.84	0.00	17.10	−0.10	0.16
		Cl	1.93	5.40	0.00	0.00	7.33	−0.33	−0.15
K_2_NaRhBr_6_	0.10%	K	2.18	6.42	0.24	0.00	8.84	0.16	0.23
		Na	2.24	6.59	0.00	0.00	8.83	0.17	0.18
		Rh	2.76	6.91	7.97	0.00	17.64	−0.64	0.10
		Br	1.64	5.33	0.00	0.00	6.98	0.02	−0.13
K_2_NaRhI_6_	0.12%	K	2.18	6.31	0.26	0.00	8.75	0.25	0.23
		Na	2.31	6.65	0.00	0.00	8.97	0.03	0.17
		Rh	2.74	7.02	8.20	0.00	17.97	−0.97	0.01
		I	1.69	5.24	0.00	0.00	6.93	0.07	−0.11

The Hirshfeld analysis shows a similar qualitative variation, with the F and I atoms carrying charges of −0.23 and −0.11, respectively. Although the absolute charge values differ between the Mulliken and Hirshfeld schemes because of their different charge-partitioning procedures, both approaches indicate systematic changes in charge distribution across the halogen series. The increasing halogen ionic radius also increases the lattice parameter and Rh–X bond distance, which can reduce orbital overlap and modify the charge distribution. The charge-spilling values of 0.10–0.12% indicate a low contribution from charge spilling in the population calculations. Overall, the population analyses provide qualitative evidence of charge transfer and changes in the ionic/covalent character across the K_2_NaRhX_6_ series.

## 4. Photocatalytic properties

The photocatalytic potential of the K_2_NaRhX_6_ series was assessed from the calculated band-edge positions relative to the water redox potentials and to the standard CO_2_ reduction levels, both referenced to the normal hydrogen electrode (NHE).

### 4.1. Hydrogen evolution

Photocatalytic water splitting is a sustainable approach for generating hydrogen (H_2_) by utilizing solar energy. The photocatalytic performance of a material can be quantitatively assessed using the calculated CBM and VBM positions. These band edge potentials are commonly estimated using the electronegativity method:^77^

18.1 2H^+^ + 2e^−^ → H_2_

18.2 2H_2_O → O_2_ + 4H^+^ + 4e^−^

18.3 2H_2_O → 2H_2_ + O_2_

For efficient water splitting, the photocatalyst must possess appropriate band gap energy and band edge alignment relative to the redox potential of water. The conduction band minimum (CBM) must lie at a potential more negative level than the hydrogen reduction level (0 eV *vs.* NHE), while the valence band maximum (VBM) must lie at a potential more positive than the oxygen evolution level (+1.23 eV *vs.* NHE).

The band edge potentials of the K_2_NaRhX_6_ (X = F, Cl, Br, I) compounds were estimated using the electronegativity approach according to the following relations:^78–80^19
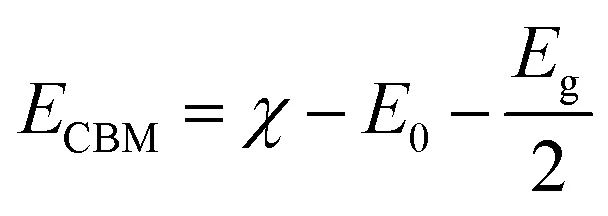
20
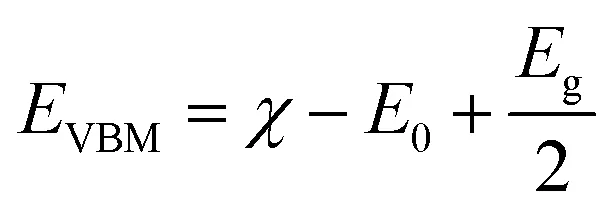
where *E*_0_ is the free-electron energy on the hydrogen scale (4.5 eV), *E*_g_ is the optical band gap, and *χ* represents the absolute electronegativity of the semiconductor. The absolute electronegativity of K_2_NaRhX_6_ compounds was estimated from the geometric mean of the constituent atomic electronegativities:^81^

21 *χ*_K_2_NaRhX_6__ = (*χ*_K_2__*χ*_Na_*χ*_Rh_*χ*_X_6__)^1/10^

The calculated band-edge positions are summarized in Table 5. It should be noted that the Mulliken electronegativity method provides a first-order estimation of band alignment and mainly reflects bulk electronic properties. This approach does not explicitly include surface effects, adsorption energetics, catalytic active sites, overpotentials, or charge-carrier recombination dynamics, which can influence practical photocatalytic performance.

**Table 5:** Calculated optical band gap (*E*_g_), and band edge positions (CBM and VBM) of K_2_NaRhX_6_ (X = F, Cl, Br, I)

Compound	*E*_g_ (eV)	CBM (eV)	VBM (eV)
K_2_NaRhF_6_	4.503	−0.551	3.952
K_2_NaRhCl_6_	2.506	−0.053	2.453
K_2_NaRhBr_6_	1.899	−0.049	1.850
K_2_NaRhI_6_	1.449	−0.024	1.425

Table 5 and Fig. 10 present the calculated CBM and VBM positions of K_2_NaRhX_6_ relative to the normal hydrogen electrode (NHE). The calculated CBM values of all investigated compounds lie below the hydrogen reduction potential, indicating that photogenerated electrons can thermodynamically participate in hydrogen evolution reactions. Among the investigated compounds, K_2_NaRhF_6_ exhibits the most negative CBM value (−0.551 eV), indicating the strongest reduction capability. In comparison, K_2_NaRhCl_6_ (−0.053 eV), K_2_NaRhBr_6_ (−0.049 eV), and K_2_NaRhI_6_ (−0.024 eV) exhibit comparatively weaker reducing potentials. On the oxidation side, all compounds possess VBM values above the oxygen evolution potential (+1.23 eV), suggesting favorable oxidation capability for water oxidation reactions. K_2_NaRhF_6_ exhibits the highest VBM value (3.952 eV), followed by K_2_NaRhCl_6_ (2.453 eV), K_2_NaRhBr_6_ (1.850 eV), and K_2_NaRhI_6_ (1.425 eV). These results indicate that K_2_NaRhF_6_ shows the most favorable band alignment for overall water splitting from a thermodynamic perspective. The chloride and bromide compounds also satisfy the required redox alignment conditions, although their smaller reduction potential may influence photocatalytic efficiency. In contrast, K_2_NaRhI_6_ exhibits comparatively weaker oxidation capability because of its lower VBM value. Optical band gaps additionally influence photocatalytic activity.

**Fig. 10:**
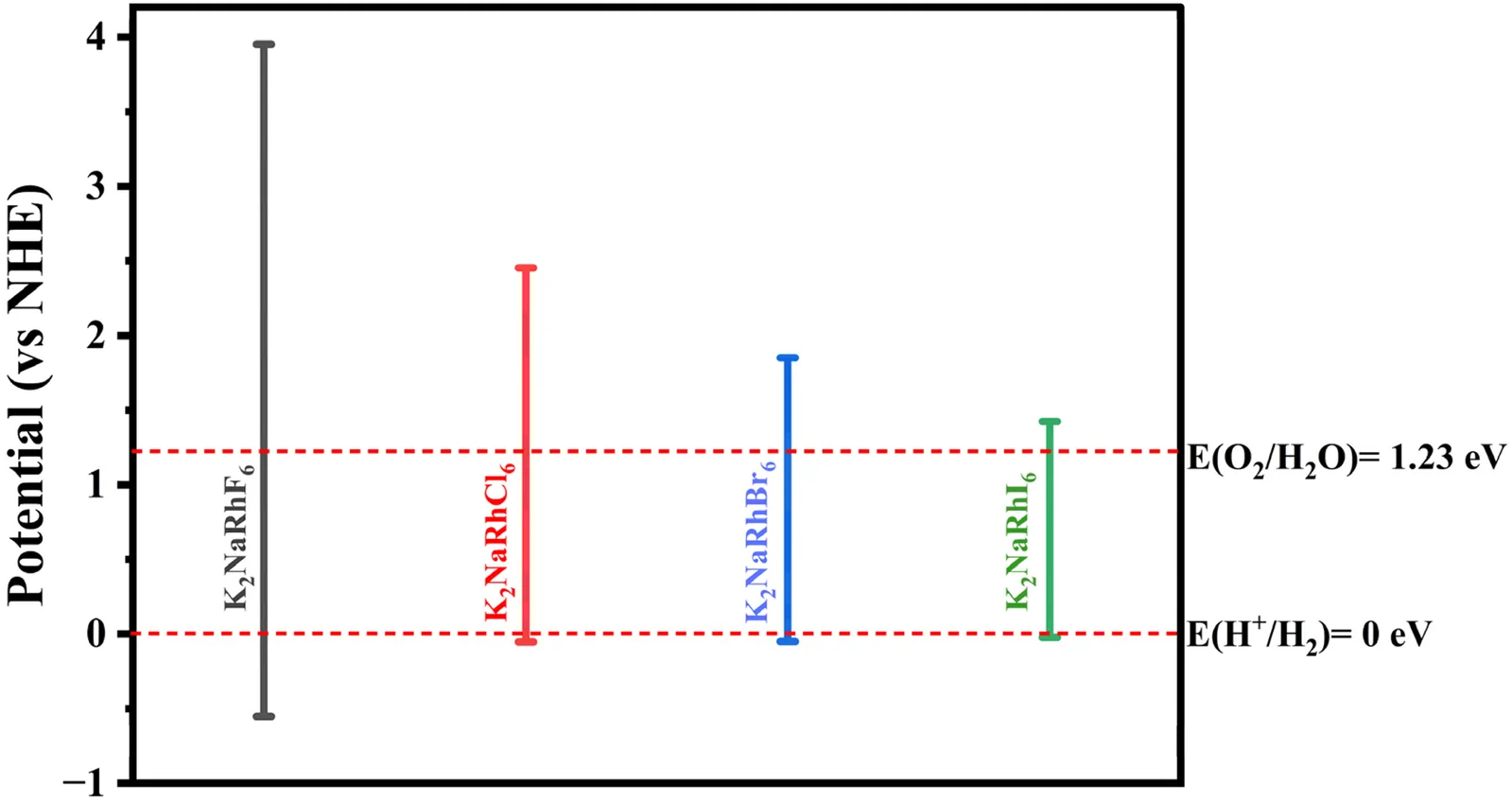
Photocatalytic band edge potentials of K_2_NaRhX_6_ relative to the Normal Hydrogen Electrode (NHE).

Although K_2_NaRhF_6_ exhibits the most favorable band alignment, its large optical band gap (4.503 eV) restricts light absorption mainly to the ultraviolet region. In comparison, K_2_NaRhCl_6_ and K_2_NaRhBr_6_ provide a more balanced combination of visible-light absorption and thermodynamic band alignment, which may be beneficial for solar-driven photocatalytic applications. Overall, the calculated band-edge alignment suggests that K_2_NaRhX_6_ double perovskites possess thermodynamically favorable electronic structures for photocatalytic water splitting at the bulk level. However, detailed photocatalytic performance additionally depends on surface reaction pathways, carrier dynamics, and catalytic kinetics, which are beyond the scope of the present study.

### 4.2. CO_2_ photo-degradation capacity

The main photocatalytic CO_2_ reduction reactions can be expressed as:CO_2_ + 8H^+^ + 8e^−^ → CH_4_ + 2H_2_OCO_2_ + 2H^+^ + 2e^−^ → HCOOHCO_2_ + 2H^+^ + 2e^−^ → CO + H_2_OCO_2_ + 6H^+^ + 6e^−^ → CH_3_OH + H_2_O

For these reactions to proceed, the conduction band minimum (CBM) of the photocatalyst must lie at a sufficiently negative potential relative to the CO_2_ reduction levels, while the valence band maximum (VBM) should support the accompanying oxidation reactions. Fig. 9 compares the calculated band-edge positions of K_2_NaRhX_6_ (X = F, Cl, Br, and I) with the standard CO_2_ reduction potential referenced to the normal hydrogen electrode (NHE). The dashed horizontal lines represent the reduction potential for CO_2_/CH_4_ (0.169 eV), CO_2_/CH_3_OH (0.017 eV), CO_2_/CO (−0.105 eV), and CO_2_/HCOOH (−0.169 eV). The calculated CBM values of the investigated compounds are positioned at more negative potentials than several of these reduction levels, indicating favorable thermodynamic driving force for selected CO_2_ reduction pathways. Among the investigated compounds, K_2_NaRhF_6_ exhibits the most negative CBM value (−0.551 eV), followed by K_2_NaRhI_6_ (−0.480 eV), K_2_NaRhCl_6_ (−0.053 eV), and K_2_NaRhBr_6_ (0.049 eV). These band positions indicate that K_2_NaRhF_6_ possesses the strongest reduction capability for CO_2_ conversion reactions. The chloride and bromide compounds also satisfy the required reduction potentials for products such as HCOOH and CO, although with a comparatively smaller driving force. In addition, the relatively higher VBM positions of K_2_NaRhF_6_ and K_2_NaRhCl_6_ provide favorable oxidation capability that can support the overall photocatalytic process. The halide composition significantly influences the electronic structure and photocatalytic characteristics of the K_2_NaRhX_6_ series. As the halogen changes from F to I, the valence band shifts toward lower potentials, reducing oxidation strength and modifying the relative band alignment. Based on the calculated band-edge positions, K_2_NaRhF_6_ exhibits the most favorable overall thermodynamic alignment for photocatalytic CO_2_ reduction, followed by K_2_NaRhCl_6_ and K_2_NaRhBr_6_, whereas K_2_NaRhI_6_ shows comparatively weaker oxidation capability. The present analysis is based on band-edge alignment obtained from the Mulliken electronegativity approach, which primarily reflects bulk electronic properties. Photocatalytic CO_2_ reduction additionally depends on surface adsorption energetics, catalytic active sites, charge-carrier recombination dynamics, and multi-electron reaction intermediates that determine product selectivity. These surface-level and kinetic effects are beyond the scope of the present work. Therefore, the calculated band-edge alignment provides a thermodynamic framework for evaluating the CO_2_ reduction potential of K_2_NaRhX_6_ double perovskites.

## 5. SCAPS-1D device modeling

The DFT calculations indicate that the K_2_NaRhX_6_ (X = F, Cl, Br, I) compounds possess several characteristics desirable for photovoltaic applications, including structural stability, suitable electronic band gaps, favorable carrier transport properties, strong optical absorption, and mechanically compliant crystal structures. These findings suggest that the investigated materials can potentially serve as absorber layers in solar-cell devices.

However, material-level properties alone are insufficient to evaluate the actual photovoltaic performance. Therefore, device-level simulations were carried out using the SCAPS-1D software to assess the photovoltaic potential of the proposed absorbers. The DFT-derived parameters, including the band gap, electron affinity, dielectric constant, effective density of states, and carrier mobilities, were employed as input parameters in the SCAPS-1D simulator (Table S1). Based on these parameters, an FTO/SnS_2_/K_2_NaRhX_6_ device architecture was constructed, as illustrated in Fig. 11. Since parameters such as absorber thickness, shallow acceptor concentration (*N*_A_), bulk defect density (*N*_t_), and interface defect density (*N*_it_) cannot be directly determined from first-principles calculations, they were systematically optimized within the SCAPS-1D framework. This optimization enables the identification of the optimum device configuration and provides an estimate of the theoretical upper-limit photovoltaic performance achievable by K_2_NaRhX_6_-based solar cells. In this way, the SCAPS-1D analysis establishes a direct connection between the intrinsic material properties predicted by DFT and the expected device-level performance.

**Fig. 11:**
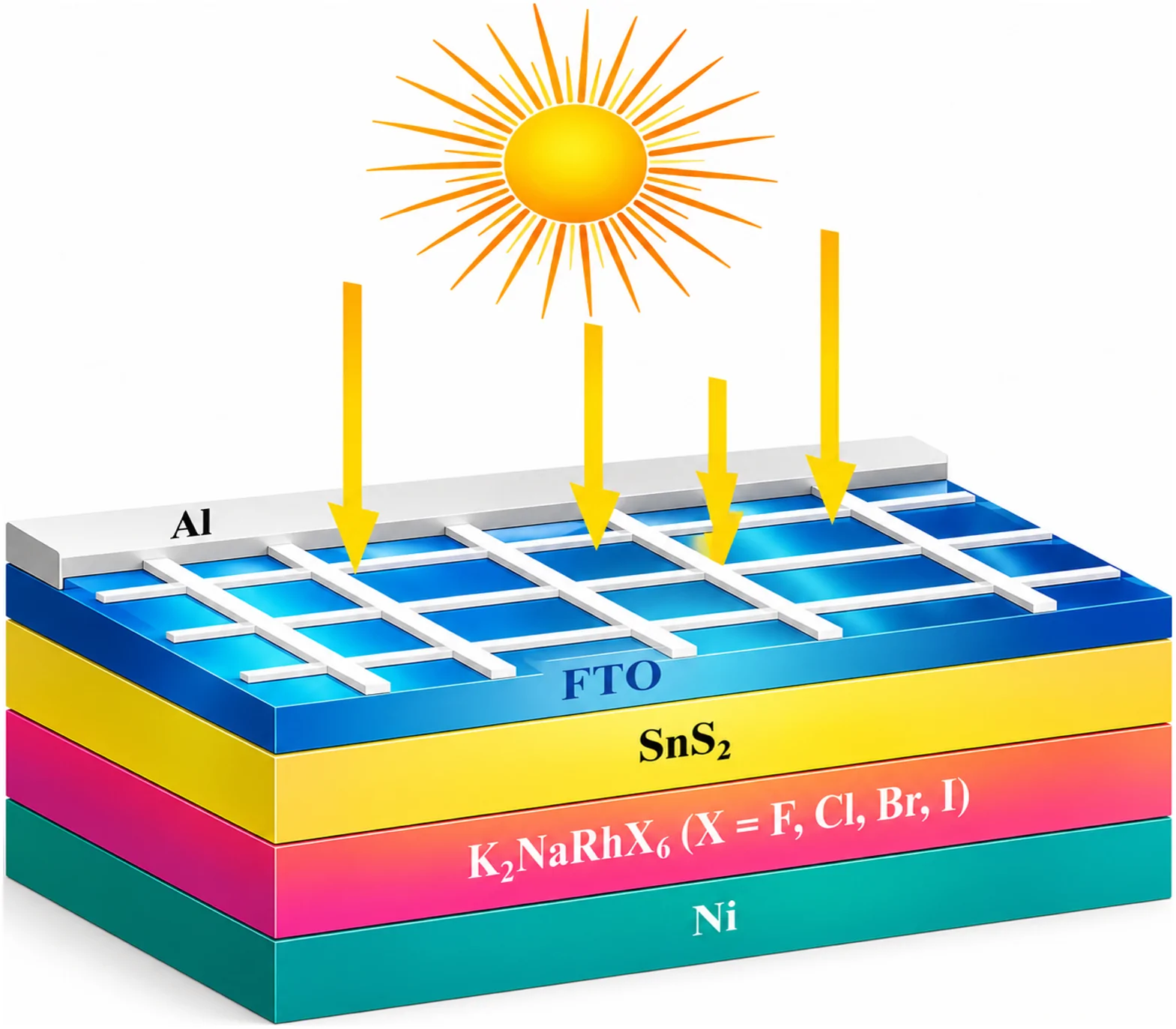
Schematic illustration of the proposed FTO/SnS_2_/K_2_NaRhX_6_ (X = F, Cl, Br, I) solar cell architecture employed in the SCAPS-1D simulations.

Table S2 summarizes the interface defect parameters used in the simulation of the SnS_2_/K_2_NaRhX_6_ solar cell structures, where X represents F, Cl, Br, and I. The interfaces considered are SnS_2_/K_2_NaRhF_6_, SnS_2_/K_2_NaRhCl_6_, SnS_2_/K_2_NaRhBr_6_, and SnS_2_/K_2_NaRhI_6_. For all interfaces, the electron and hole capture cross-sections are fixed at 1 × 10^−19^ cm^2^. This low capture cross-section value indicates a reduced probability of carrier trapping and recombination at the heterojunction interface, which is beneficial for efficient charge transport and enhanced photovoltaic performance. The defect type for each interface is assumed to be neutral, meaning that the interface defects do not introduce charged states that could significantly alter the internal electric field distribution within the device. Additionally, the total interface defect density is maintained at 1 × 10^11^ cm^−2^ for all SnS_2_/K_2_NaRhX_6_ interfaces. This relatively low interface defect density suggests good interfacial quality and minimized recombination losses at the junction between the SnS_2_ electron transport layer and the K_2_NaRhX_6_ absorber layers.

Fig. S2(a–d) illustrates the energy band alignment diagrams of the FTO/SnS_2_/K_2_NaRhX_6_ heterostructure solar cells for different halide compositions, where X = F, Cl, Br, and I. The plots show the variation of the conduction band edge (*E*_C_), valence band edge (*E*_V_), electron quasi-Fermi level (Fn), and hole quasi-Fermi level (Fp) across the device thickness. In all configurations, the device consists of a 0.05 µm FTO window layer, a 0.05 µm SnS_2_ electron transport layer, and a 0.70 µm K_2_NaRhX_6_ absorber layer. The energy band diagrams confirm proper heterojunction formation between the transport layer and absorber, facilitating effective charge separation and carrier transport. Fig. S2(a) corresponds to the K_2_NaRhF_6_ absorber with a band gap of 1.861 eV. The relatively wide band gap results in a larger separation between the conduction and valence bands, which is advantageous for achieving a high open-circuit voltage but may limit light absorption in the longer wavelength region. Fig. S2(b), the K_2_NaRhCl_6_ absorber exhibits a reduced band gap of 1.267 eV. The narrower energy gap improves visible-light absorption while maintaining suitable band alignment with the SnS_2_ layer for electron extraction. Fig. S2(c) shows the band structure for K_2_NaRhBr_6_ with a band gap of 0.936 eV. The further reduction in band gap enhances photon absorption over a broader spectral range, which can contribute to higher photocurrent generation. Fig. S2(d) presents the K_2_NaRhI_6_-based device, which possesses the smallest band gap of 0.579 eV. The conduction and valence bands become significantly closer, indicating strong absorption capability in the visible and near-infrared regions. Additionally, the quasi-Fermi levels remain well separated under operation, suggesting efficient carrier generation and transport.

### 5.1. Thickness-dependent performance optimization of K_2_NaRhX_6_ solar cells

Fig. S3(a–d) illustrates the influence of absorber layer thickness on the photovoltaic performance of the SnS_2_/K_2_NaRhX_6_ (X = F, Cl, Br, I) solar cells. The absorber thickness was varied from 0.3 to 2.5 µm to investigate its effect on the *V*_OC_, *J*_SC_, FF, and PCE. Since absorber thickness directly controls light absorption, carrier generation, and charge transport, its optimization is crucial for achieving high photovoltaic performance.^82^ As shown in Fig. S3(a), the *V*_OC_ behavior strongly depends on the absorber composition. The K_2_NaRhF_6_-based device exhibits a slight increase in *V*_OC_ from 0.925 V at 0.3 µm to 0.937 V at 0.7 µm, followed by saturation near 0.939 V at larger thicknesses. In contrast, K_2_NaRhCl_6_, K_2_NaRhBr_6_, and K_2_NaRhI_6_ show a continuous reduction in *V*_OC_ with increasing thickness. For example, the *V*_OC_ of K_2_NaRhCl_6_ decreases from 0.929 V to 0.890 V, while K_2_NaRhBr_6_ and K_2_NaRhI_6_ decline from 0.625 to 0.569 V and from 0.407 to 0.362 V, respectively. This reduction can be attributed to enhanced bulk recombination within thicker absorber layers, which increases the probability of photogenerated carriers recombining before collection. Fig. S3(b) presents the variation of *J*_SC_ with absorber thickness. Unlike *V*_OC_, the current density increases rapidly as the thickness increases because a larger absorber layer captures more incident photons and generates a greater number of electron–hole pairs. For K_2_NaRhCl_6_, *J*_SC_ rises from 29.91 mA cm^−2^ at 0.3 µm to 34.88 mA cm^−2^ at 0.7 µm, corresponding to an increase of approximately 16.6%. Similarly, K_2_NaRhBr_6_ increases from 43.90 to 49.46 mA cm^−2^, while K_2_NaRhI_6_ increases from 58.60 to 62.19 mA cm^−2^. However, beyond 0.7 µm, the improvement becomes much smaller. For instance, increasing the thickness from 0.7 to 2.5 µm enhances the *J*_SC_ of K_2_NaRhCl_6_ by only about 3.9%, indicating that most useful photons are already absorbed within the first 0.7 µm of the absorber layer. The effect of absorber thickness on FF is shown in Fig. S3(c). The FF remains relatively stable over the investigated thickness range, suggesting that carrier collection efficiency and resistive losses are only weakly affected by thickness. At 0.7 µm, FF values of 79.15%, 83.23%, 82.27%, and 76.15% are obtained for K_2_NaRhF_6_, K_2_NaRhCl_6_, K_2_NaRhBr_6_, and K_2_NaRhI_6_, respectively. Although K_2_NaRhCl_6_ shows a gradual increase in FF with thickness, the variations for all devices remain within a narrow range, indicating stable charge-transport characteristics. The combined influence of *V*_OC_, *J*_SC_, and FF on the device efficiency is presented in Fig. S3(d). The PCE initially increases sharply with thickness due to the substantial enhancement in current generation. For example, the efficiency of K_2_NaRhCl_6_ increases from 22.81% at 0.3 µm to 26.64% at 0.7 µm, corresponding to an improvement of approximately 16.8%. Similarly, K_2_NaRhF_6_ improves from 9.55% to 12.07%, while K_2_NaRhBr_6_ increases from 22.55% to 24.53%. However, after 0.7 µm, the efficiency gain becomes marginal despite the continued increase in absorber thickness. For K_2_NaRhCl_6_, increasing the thickness from 0.7 to 1.6 µm improves the efficiency by only 0.67% (26.64% to 27.32%). Likewise, K_2_NaRhBr_6_ exhibits only a 0.15% increase in efficiency between 0.7 and 1.0 µm before gradually declining. More importantly, the K_2_NaRhI_6_-based device achieves its highest efficiency near the lower-thickness region and subsequently experiences a continuous reduction in PCE due to increasing recombination losses. Therefore, although slightly higher efficiencies can be achieved at larger thicknesses for some absorbers, the additional gain is very small compared with the significant increase in material consumption and the higher probability of carrier recombination. Consequently, an absorber thickness of 0.7 µm was selected as the optimized value for all subsequent simulations. At this thickness, the devices already achieve more than 97–99% of their maximum attainable efficiencies while requiring substantially less absorber material. Thus, 0.7 µm provides the best compromise between photovoltaic performance, material utilization, and practical device fabrication, making it the most suitable thickness for further optimization studies.

### 5.2. Effect of total defect density and shallow acceptor density

Fig. S4(a) illustrates the influence of total defect density (*N*_t_) on the photovoltaic performance of SnS_2_/K_2_NaRhX_6_ solar cells. The defect density was varied from 10^10^ to 10^18^ cm^−3^ to evaluate its impact on *V*_OC_, *J*_SC_, FF, and PCE. The overall device performance deteriorates with increasing defect density. As shown in Fig. S4(a), the photovoltaic parameters remain nearly unchanged between 10^10^ and 10^14^ cm^−3^, indicating that recombination losses are negligible within this low-defect regime. However, beyond 10^14^ cm^−3^, a significant degradation in performance is observed for all devices. For example, the PCE of the K_2_NaRhCl_6_-based solar cell decreases from 26.64% at 10^14^ cm^−3^ to 10.76% at 10^18^ cm^−3^. Similarly, the efficiency of K_2_NaRhBr_6_ drops from about 24.525% to below 7.34%, while K_2_NaRhI_6_ exhibits the most pronounced degradation, decreasing from 18.53% to 5.33%. The reduction in efficiency is accompanied by substantial decreases in FF and *V*_OC_, confirming that defect-assisted recombination becomes increasingly dominant at higher defect concentrations. In contrast, *J*_SC_ remains relatively stable up to approximately 10^16^ cm^−3^ and then decreases gradually, suggesting that increasing defect density primarily affects carrier recombination and collection rather than photon absorption. Since all photovoltaic parameters remain close to their optimum values around 10^14^ cm^−3^, while higher defect concentrations lead to severe performance losses, a total defect density of 10^14^ cm^−3^ was selected as the optimized value for subsequent simulations. This value provides a suitable balance between device performance and realistic absorber quality.

Fig. S4(b) presents the effect of shallow acceptor density (*N*_A_) on the photovoltaic characteristics of the investigated solar cells. The acceptor concentration was varied from 10^13^ to 10^20^ cm^−3^. Increasing the acceptor density enhances the built-in electric field across the junction, improves charge separation, and suppresses carrier recombination, thereby increasing both *V*_OC_ and FF. For instance, the *V*_OC_ of the K_2_NaRhCl_6_-based device increases from approximately 0.86 V at 10^13^ cm^−3^ to about 0.96 V at 10^20^ cm^−3^, while its FF increases from nearly 78% to 85%. The PCE initially improves with increasing acceptor concentration owing to enhanced carrier extraction and reduced recombination losses. For K_2_NaRhCl_6_, the efficiency increases from 23.76% at 10^13^ cm^−3^ to around 26.64% at 10^17^ cm^−3^. A similar trend is observed for K_2_NaRhBr_6_, where the PCE increases continuously from about 19.801% to over 32.45% with increasing acceptor density. However, excessively high doping concentrations can adversely affect device performance. This behavior is particularly evident for K_2_NaRhI_6_, whose efficiency remains nearly constant up to 10^17^ cm^−3^ and then decreases sharply beyond 10^19^ cm^−3^, accompanied by significant reductions in FF and *J*_SC_. Such deterioration can be attributed to increased impurity scattering, enhanced carrier recombination, and reduced carrier transport efficiency at very high doping concentrations. Although certain devices exhibit slight efficiency improvements beyond 10^17^ cm^−3^, the performance gain becomes relatively small compared with the substantial increase in doping concentration. Moreover, excessively high acceptor densities may be difficult to achieve experimentally and can introduce additional recombination pathways. Therefore, a shallow acceptor density of 10^17^ cm^−3^ was selected as the optimized value for all subsequent simulations. At this concentration, all K_2_NaRhX_6_ absorbers exhibit stable operation, efficient carrier separation, and near-optimum photovoltaic performance, making it the most appropriate choice for further device optimization studies.

### 5.3. The effect of temperature

Fig. S5(a–d) illustrates the effect of operating temperature on the photovoltaic performance parameters of FTO/SnS_2_/K_2_NaRhX_6_ solar cells over the temperature range of 280 to 450 K. The circular plots represent the variations in *V*_OC_, *J*_SC_, FF and PCE for different absorber materials. Fig. S5(a) shows the variation of *V*_OC_ with temperature. The open-circuit voltage decreases continuously as the temperature increases for all absorber materials. This reduction is mainly caused by the increase in intrinsic carrier concentration and enhanced recombination at elevated temperatures. K_2_NaRhCl_6_ exhibits the highest *V*_OC_, while K_2_NaRhI_6_ shows comparatively lower voltage values due to its narrower band gap. Fig. S5(b) presents the effect of temperature on the short-circuit current density (*J*_SC_). In contrast to *V*_OC_, the current density increases gradually with rising temperature. Higher temperatures enhance carrier generation and improve photon absorption, resulting in increased photocurrent. K_2_NaRhI_6_ demonstrates the highest *J*_SC_ values because of its small band gap and superior light absorption capability. Fig. S5(c) illustrates the variation of power conversion efficiency (PCE) with temperature. Efficiency decreases significantly as temperature increases for all devices. This decline is mainly attributed to the strong reduction in *V*_OC_ and the increase in recombination losses at higher temperatures. Among the investigated absorbers, K_2_NaRhCl_6_ maintains the highest efficiency throughout the temperature range, indicating better thermal stability. Fig. S5(d) shows the influence of temperature on the fill factor (FF). The FF decreases progressively with increasing temperature for all devices, suggesting increased series resistance and recombination losses at elevated operating temperatures. The reduction is more pronounced in the K_2_NaRhI_6_-based device, while K_2_NaRhCl_6_ retains relatively higher FF values.

### 5.4. Current density–voltage (*J*–*V*) and external quantum efficiency (QE)

Fig. S6(a) presents the current density–voltage (*J*–*V*) characteristics of FTO/SnS_2_/K_2_NaRhX_6_ (X = F, Cl, Br, and I) solar cells under illumination conditions. The curves demonstrate the photovoltaic behavior of the devices by showing the relationship between current density and applied voltage. Among the investigated absorbers, K_2_NaRhI_6_ exhibits the highest *J*_SC_, reaching approximately 62 mA cm^−2^, due to its narrow band gap and enhanced light absorption capability. However, its *V*_OC_ is comparatively lower. In contrast, the K_2_NaRhF_6_-based device shows the highest *V*_OC_ but the lowest current density because of its wider band gap and limited absorption range. K_2_NaRhCl_6_ and K_2_NaRhBr_6_ demonstrate balanced photovoltaic performance with moderate voltage and high current density values, indicating efficient charge generation and collection. Fig. S6(b) illustrates the external quantum efficiency (QE) spectra of the devices as a function of wavelength. The QE curves indicate the ability of the solar cells to convert incident photons into collected charge carriers across the solar spectrum. All devices exhibit high QE values in the visible region, approaching nearly 100% at shorter wavelengths. The absorption edge shifts toward longer wavelengths from K_2_NaRhF_6_ to K_2_NaRhI_6_ as the band gap decreases. K_2_NaRhI_6_ shows the broadest spectral response, extending beyond 2100 nm, confirming its superior near-infrared light absorption capability. Conversely, K_2_NaRhF_6_ displays the shortest cutoff wavelength due to its larger band gap. The progressive redshift in the QE spectra demonstrates the tunability of the optical absorption properties through halide substitution in the K_2_NaRhX_6_ absorber materials.

### 5.5. Photovoltaic performance analysis

Fig. S7 summarizes the optimized photovoltaic performance parameters of the FTO/SnS_2_/K_2_NaRhX_6_ solar cells obtained under the optimized device conditions (*t* = 0.7) µm, (*N*_A_ = 10^17^ cm^−3^, and *N*_t_ = 10^14^ cm^−3^). The comparison includes the PCE, *V*_OC_, *J*_SC_, and FF for each absorber material. Among the investigated absorbers, K_2_NaRhCl_6_ exhibits the highest PCE of 26.64%, followed by K_2_NaRhBr_6_ of 24.53%, K_2_NaRhI_6_ of 18.53%, and K_2_NaRhF_6_ of 12.07%. The superior performance of K_2_NaRhCl_6_ originates from its balanced photovoltaic characteristics, including a relatively high *V*_OC_ of 0.918 V, a large *J*_SC_ of 34.89 mA cm^−2^, and an FF of 83.23%. In contrast, although K_2_NaRhBr_6_ delivers a significantly higher *J*_SC_ of 49.46 mA cm^−2^, its lower *V*_OC_ of 0.603 V limits the overall efficiency. Similarly, K_2_NaRhI_6_ achieves the highest current density of 62.19 mA cm^−2^ because of its narrow band gap and extended spectral absorption, but its relatively low *V*_OC_ of 0.391 V and FF of 76.15% reduce the final PCE. On the other hand, K_2_NaRhF_6_ exhibits the highest *V*_OC_ of 0.937 V owing to its wider band gap; however, its lower *J*_SC_ of 16.28 mA cm^−2^ restricts the achievable efficiency. The observed trends demonstrate the fundamental trade-off between voltage and current generation in photovoltaic absorbers. As the band gap decreases from F to I, *J*_SC_ increases substantially due to enhanced photon absorption, whereas *V*_OC_ decreases because of the reduced band-gap energy. Consequently, K_2_NaRhCl_6_ provides the most favorable balance between these competing parameters, resulting in the highest overall device performance.

It should be noted that these efficiencies represent the theoretical upper-limit performance predicted by SCAPS-1D simulations under idealized assumptions, including optimized material quality, controlled defect densities, and ideal interface properties. Therefore, the reported efficiencies should be considered as performance benchmarks rather than experimentally achievable values. From the perspective of the Shockley–Queisser (SQ) limit, the obtained efficiencies are physically reasonable. The K_2_NaRhCl_6_ absorber, with a DFT-derived band gap of approximately 1.27 eV, lies close to the optimum band-gap range for single-junction solar cells (≈1.1–1.4 eV), where the SQ limit reaches about 30–33% under standard illumination conditions. Consequently, the predicted efficiency of 26.64% corresponds to roughly 80–88% of the theoretical SQ limit, indicating excellent photovoltaic potential.

Likewise, K_2_NaRhBr_6_ exhibits a competitive efficiency because of its strong current generation, whereas the wider-band-gap K_2_NaRhF_6_ and the very narrow-band-gap K_2_NaRhI_6_ suffer from current- and voltage-related limitations, respectively. To further evaluate the competitiveness of the proposed absorber, the optimized performance of K_2_NaRhCl_6_ was compared with several recently reported lead-free double-perovskite solar cells. Previously reported devices based on Cs_2_AgInBr_6_, Cs_2_AgBiBr_6_, Rb_2_LiGaI_6_, Cs_2_NaAlI_6_, Cs_2_AuBiCl_6_, Na_2_InAgCl_6_, and Na_2_AuGaBr_6_ achieved PCE values of 26.56%, 26.89%, 26.48%, 25.98%, 21.16%, 25.78%, and 28.96%, respectively, as shown in Table 6. The optimized K_2_NaRhCl_6_ device with a PCE of 26.64% is therefore comparable to the majority of recently proposed lead-free double-perovskite absorbers and exceeds several previously reported systems. Although its efficiency remains slightly lower than that of the highest-performing Na_2_AuGaBr_6_-based device, the overall photovoltaic performance demonstrates that K_2_NaRhCl_6_ is a highly competitive absorber material. The favorable balance between *V*_OC_, *J*_SC_, and FF, together with its mechanically stable, optically active, and electronically suitable nature, highlights its potential for future high-efficiency lead-free photovoltaic applications.

**Table 6:** Previously reported double perovskite materials performances

Devices	*V*_OC_ (V)	*J*_SC_ (mA cm^−2^)	FF (%)	PCE (%)	Ref.
FTO/IGZO/Cs_2_AgInBr_6_/CBTS/Au	1.16	27.28	83.30	26.56	83
ITO/WS_2_/Dy_2_NiMnO_6_/CFTS/Au	0.741	44.679	80.68	26.72	84
TiO_2_/Rb_2_LiGaI_6_/Cu_2_O	0.89	31.87	79.00	26.48	85
FTO/AZO/Cs_2_AgBiBr_6_/ZnTe	1.50	21.35	83.87	26.89	86
TO/ZnO/Cs_2_AuBiCl_6_/CuSbS_2_	0.68	39.94	77.72	21.16	87

## 6. Conclusion

A comprehensive first-principles and device-level investigation of K_2_NaRhX_6_ (X = F, Cl, Br, and I) double perovskites has been carried out to assess their suitability for renewable-energy applications. The calculated structural parameters, negative formation enthalpies, and tolerance-factor analysis support the energetic and geometrical feasibility of the investigated compounds within the cubic *Fm*3̄*m* double-perovskite framework. Electronic structure calculations reveal direct band-gap semiconducting behavior with systematically tunable band gaps ranging from 1.861 eV to 0.579 eV as the halogen changes from F to I. The reduction in band gap originates from enhanced Rh-d and halogen-p orbital hybridization and increasing lattice expansion. The optical analysis demonstrates strong photon absorption, tunable dielectric response, moderate reflectivity, and favorable optical conductivity across the ultraviolet-visible region. Mechanical-property calculations confirm the mechanical stability of all compounds according to the cubic Born stability criteria, while K_2_NaRhBr_6_ and K_2_NaRhI_6_ exhibit softer and more ductile behavior that is advantageous for thin-film processing and defect tolerance. Phonon calculations reveal low-frequency imaginary modes, particularly near the Brillouin-zone boundaries, indicating lattice softening and a possible tendency toward local octahedral distortions. The acoustic branches do not reach exactly zero at the *Γ* point, suggesting incomplete low-frequency convergence; therefore, the present results provide a qualitative assessment of the lattice dynamics rather than definitive evidence of dynamical stability. Moreover, the calculated band-edge positions suggest that the investigated materials possess suitable redox potentials for photocatalytic water-splitting applications. To bridge the gap between material properties and device performance, DFT-derived electronic parameters were employed in SCAPS-1D simulations. Through systematic optimization of absorber thickness, shallow acceptor concentration, and defect densities, an optimal configuration consisting of 0.7 µm absorber thickness, 10^17^ cm^−3^ acceptor density, and 10^14^ cm^−3^ defect density was identified. Under these conditions, the K_2_NaRhCl_6_-based solar cell delivered the highest photovoltaic performance with a PCE of 26.64%, *V*_OC_ of 0.918 V, *J*_SC_ of 34.89 mA cm^−2^, and FF of 83.23%, while K_2_NaRhBr_6_ achieved a competitive efficiency of 24.53%. These simulated efficiencies represent theoretical upper-limit performances predicted under optimized device conditions and indicate strong photovoltaic potential. Overall, the combined DFT, photocatalytic, and SCAPS-1D analyses demonstrate that halogen engineering provides an effective route for tailoring the multifunctional properties of K_2_NaRhX_6_ double perovskites. Among the investigated compositions, K_2_NaRhCl_6_ emerges as the most promising photovoltaic absorber, whereas K_2_NaRhBr_6_ offers an attractive balance between optical activity, carrier transport, mechanical flexibility, and energy-conversion performance. The present study establishes K_2_NaRhX_6_ double perovskites as promising lead-free candidates for future high-efficiency photovoltaic and photocatalytic technologies and provides a theoretical foundation for their experimental realization.

## Author contributions

Imtiaz Ahamed Apon, Gargee Mollika Suchi, Rifat Rafiu, Md. Azizur Rahman: investigation, conceptualization, methodology, data curation, software, supervision, validation, resource, writing original manuscript, review – editing. Samar O. Aljazzar, Jehan Y. Al-Humaidi, Mohamed Benghanem, Safwat A. Mahmoud: writing original manuscript, supervision, software, formal analysis, data curation, visualization, validation.

## Conflicts of interest

There are no conflicts to declare.

## Supplementary Material

RA-016-D6RA07563K-s001

## Data Availability

Data will be made available on reasonable request. Supplementary information (SI) is available. See DOI: https://doi.org/10.1039/d6ra07563k.
