# Supplementary material for: First-principles discovery of multifunctional K2NaRhX6 (X = F, Cl, Br, I) halide double perovskites for photovoltaic and photocatalytic applications
Source: RSC Adv. 2026 Sep 30. Online ahead of print. doi: 10.1039/d6ra07563k (PMC13626154; doi:10.1039/d6ra07563k)
Supplement: Supplementary file 1 [file RA-OLF-D6RA07563K-s001.pdf]

Supplementary Information (SI) for RSC Advances.
This journal is © The Royal Society of Chemistry 2026
Supplementary Information (SI) for RSC Advances.
This journal is © The Royal Society of Chemistry

First-Principles Discovery of Multifunctional K2NaRhX6 (X = F, Cl, Br, I) Halide Double Perovskites for Photovoltaic and Photocatalytic Application

Imtiaz Ahamed Apon[1,2], Gargee Mollika Suchi[3], Rifat Rafiu[3], Md. Azizur Rahman[4*], Samar O. Aljazzar[5], Jehan Y. Al-Humaidi[5], Mohamed Benghanem[6], Safwat A. Mahmoud[7]

[1]Electronics and Information Technology, University of South Wales, Treforest, Pontypridd, CF37 1DL, UK.

[2]Department of Electrical and Electronic Engineering, Bangladesh Army University of Science and Technology (BAUST), Saidpur-5311, Bangladesh.

[3]Department of Electrical and Electronic Engineering, Begum Rokeya University, Rangpur, 5400, Bangladesh.

[4]Department of Material Science and Engineering, Khulna University of Engineering & Technology (KUET), Khulna -9203, Bangladesh.

[5]Department of Chemistry College of Science Princess Nourah bint Abdulrahman University, P.O. Box 84428, Riyadh 11671, Saudi Arabia.

[6]Physics Department, Faculty of Science, Islamic University of Madinah, Madinah 42351, Saudi Arabia.

[7]Center for Scientific Research and Entrepreneurship, Northern Border University, Arar 73213, Saudi Arabia.

Corresponding Author's Email: *azizurrahmanatik49@gmail.com (Md. Azizur Rahman)

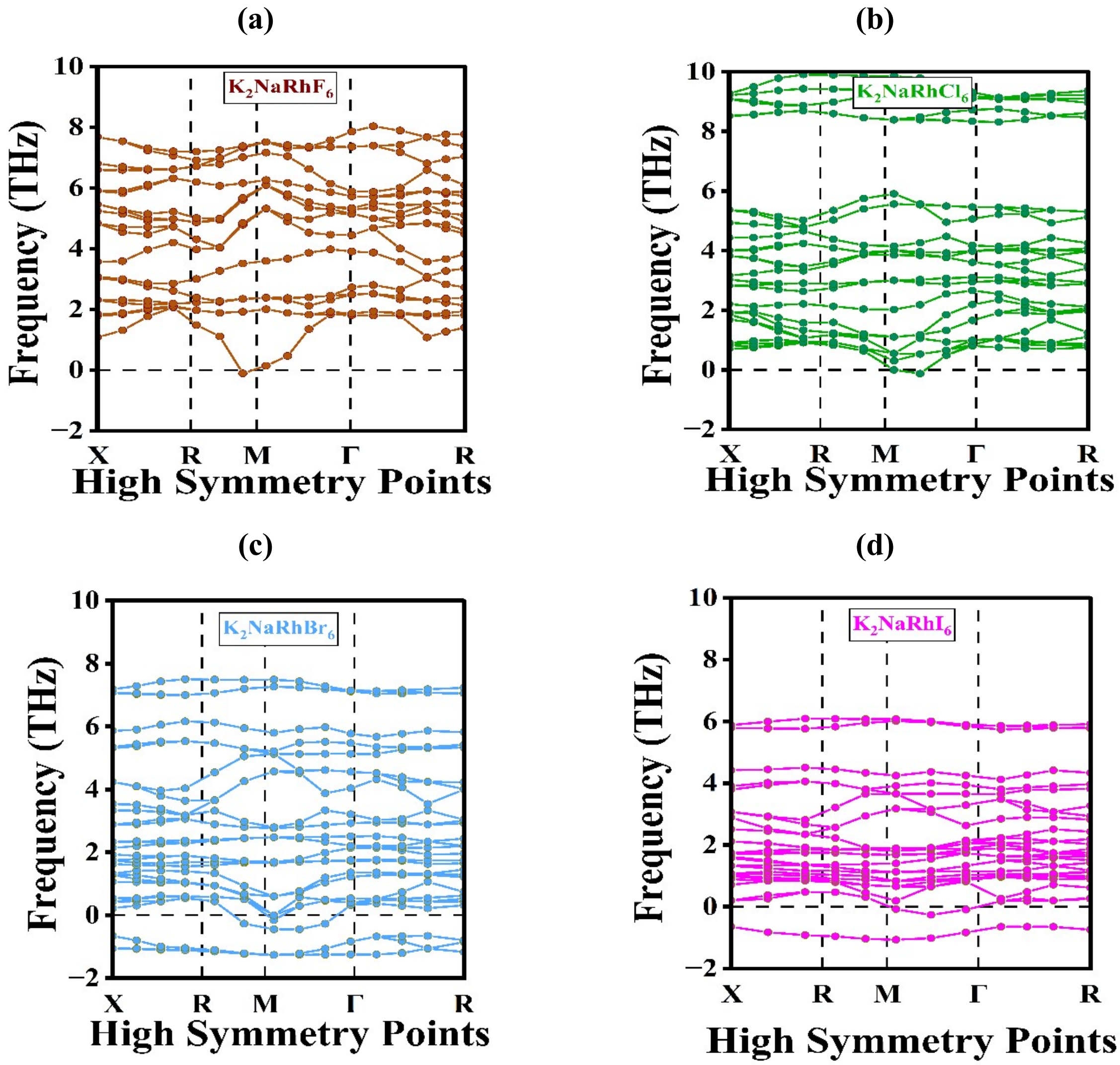

Figure S1. Phonon dispersion analysis of (a) K_2NaRhF_6, (b) $K_2NaRhCl_6$, (c) $K_2NaRhBr_6$, and (d) K_2NaRhI_6 compounds

Table S1. Physical, electronic, and defect parameters of FTO, CdS, and absorber layers (K_2NaRhX_6) used in the photovoltaic device structure.

**Parameters**	**FTO**[1–3]	**SnS_2** [1–3]	**K_2NaRhF_6**	**$K_2NaRhCl_6$**	**$K_2NaRhBr_6$**	**K_2NaRhI_6**
Thickness (nm)	50	50	700	700	700	700
Band gap, E_g (eV)	3.6	2.24	1.861	1.267	0.936	0.579
Electron affinity, χ (eV)	4.5	4.24	3.239	3.833	4.164	4.521
Dielectric permittivity, ϵ_r	10	10	2.353	3.696	4.579	6.428
C_B effective density of states, N_C (cm^{-3})	2×10^{18}	2.2×10^{18}	8.43×10^{18}	1.007×10^{19}	1.238×10^{19}	1.247×10^{19}
V_B effective density of states, N_V (cm^{-3})	1.8×10^{19}	1.8×10^{19}	1.512×10^{19}	1.515×10^{19}	1.515×10^{19}	1.503×10^{19}
Donor density, N_D (cm^{-3})	1×10^{18}	1×10^{17}	0	0	0	
Acceptor density, N_A (cm^{-3})	0	0	1×10^{17}	1×10^{17}	1×10^{17}	1×10^{17}
Electron mobility, μ_n (cm^2 V^{-1} s^{-1})	50	50	95	90	90	120
Hole mobility, μ_h (cm^2 V^{-1} s^{-1})	20	50	75	70	70	80
Defect density, N_t (cm^{-3})	1×10^{14}	1×10^{14}	1×10^{14}	1×10^{14}	1×10^{14}	1×10^{14}

Table S2. The simulated design of CdS/K_2NaRhX_6 solar cells incorporates specific interface input parameters.

**Interfaces**	**Capture Cross section: electrons/holes (cm^2)**	**Defect type**	**Total Interface defect density (cm^{-2})**
SnS_2/ K_2NaRhF_6	1×10^{-19}	neutral	1×10^{11}
$SnS_2/K_2NaRhCl_6$	1×10^{-19}	neutral	1×10^{11}
$SnS_2/K_2NaRhBr_6$	1×10^{-19}	neutral	1×10^{11}
SnS_2/K_2NaRhI_6	1×10^{-19}	neutral	1×10^{11}

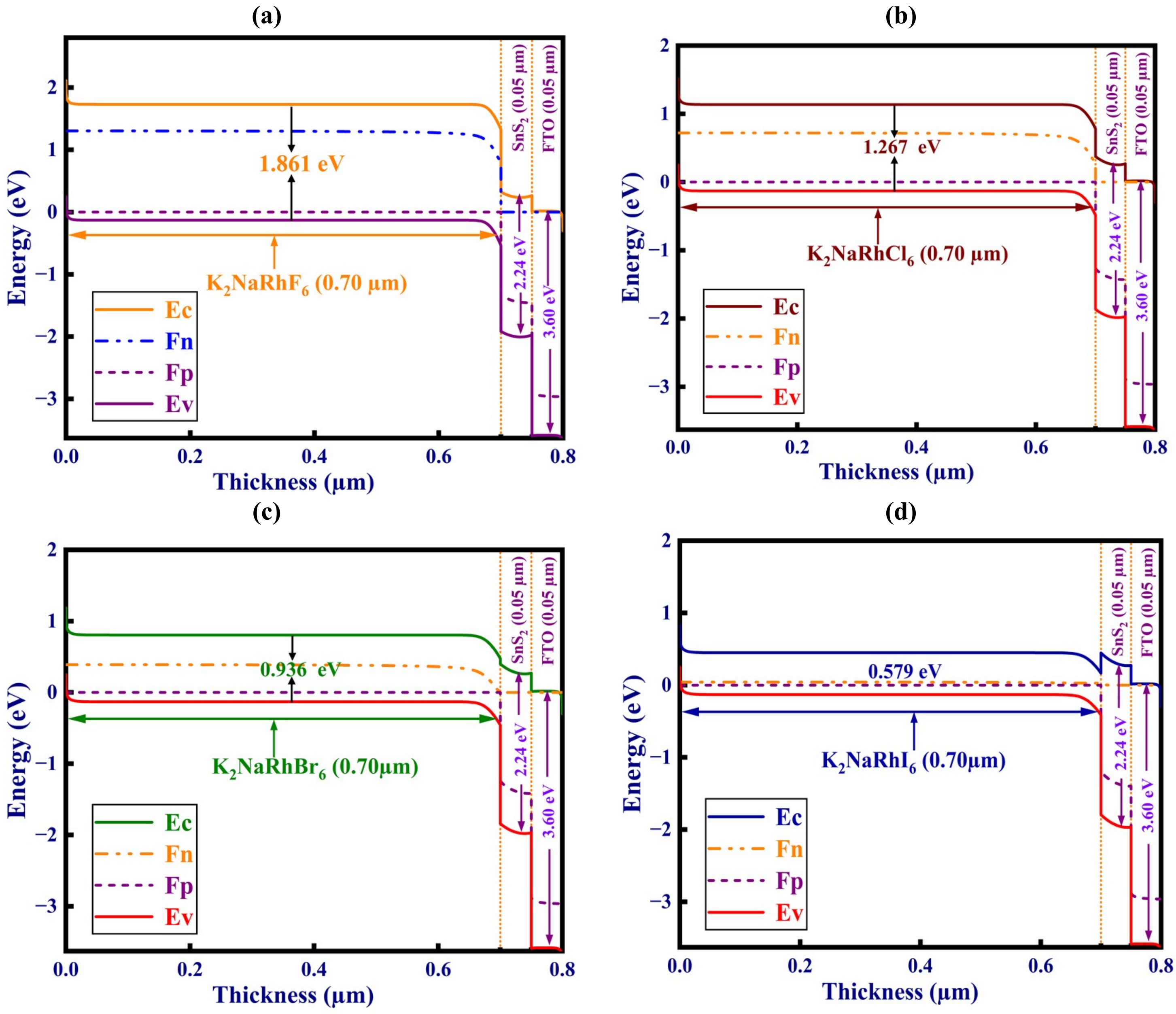

Figure S2. Energy band alignment diagrams of $CdS/SnS_2/K_2NaRhX_6$ (X = F, Cl, Br, and I) solar cells for (a) K_2NaRhF_6, (b) $K_2NaRhCl_6$, (c) $K_2NaRhBr_6$, and (d) K_2NaRhI_6 absorber layers.

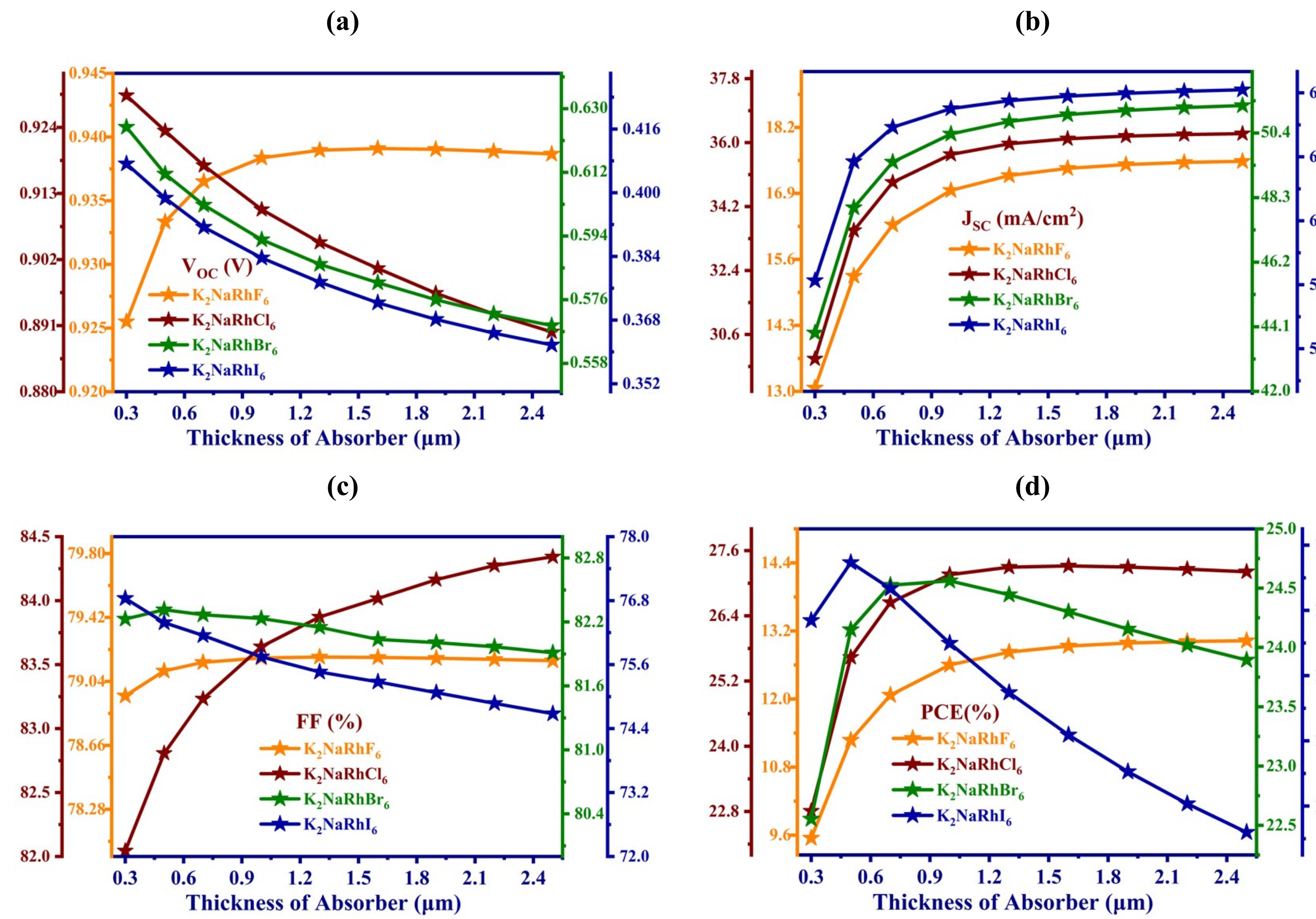

Figure S3. Variation of the photovoltaic performance parameters of $CdS/SnS_2/K_2NaRhX_6$ (X = F, Cl, Br, and I) solar cells with absorber layer thickness: (a) V_{OC}, (b) J_{SC}, (c) FF, and (d) PCE.

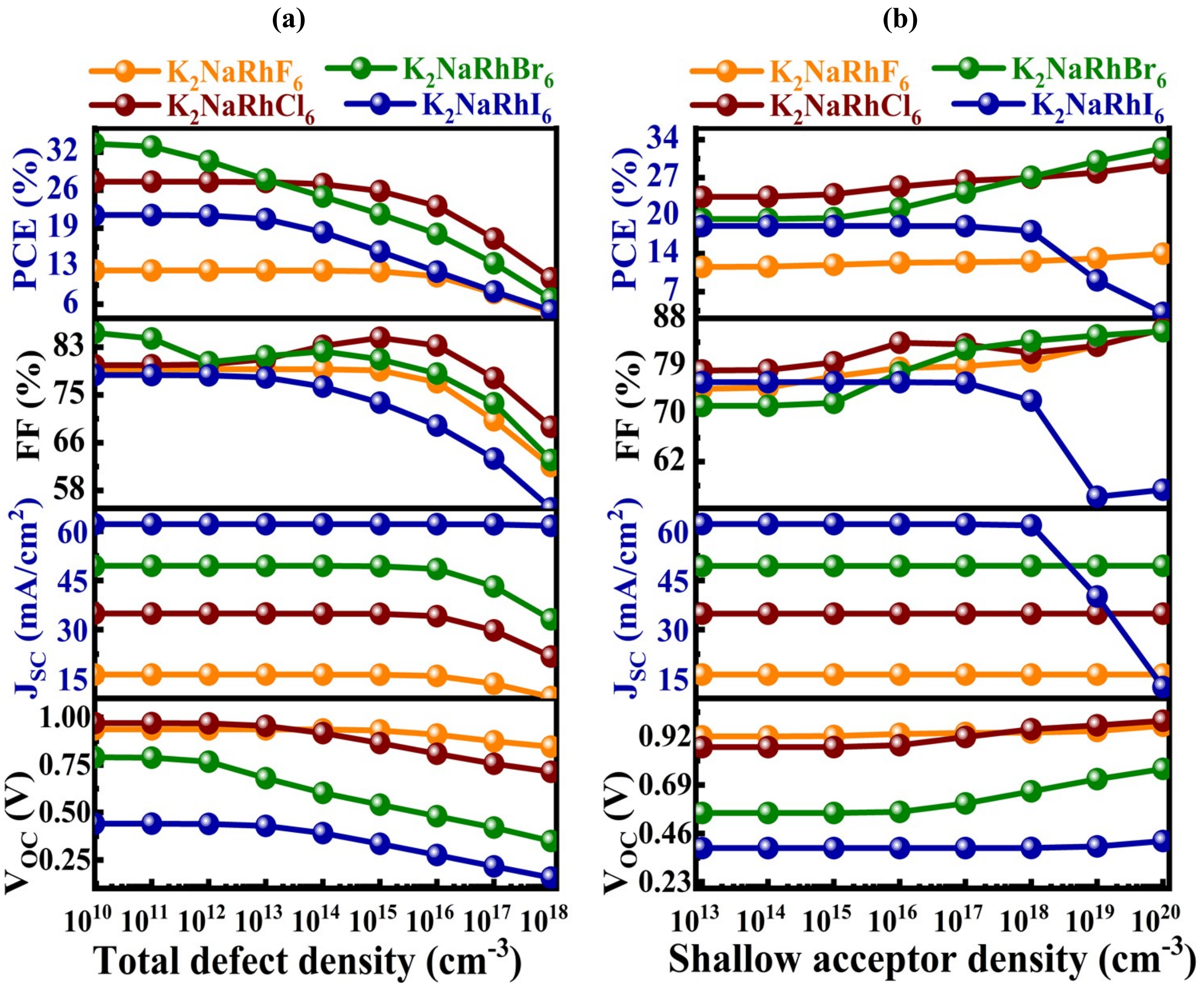

Figure S4. Effect of total defect density and acceptor density on the photovoltaic performance of $CdS/SnS_2/K_2NaRhX_6$ solar cells.

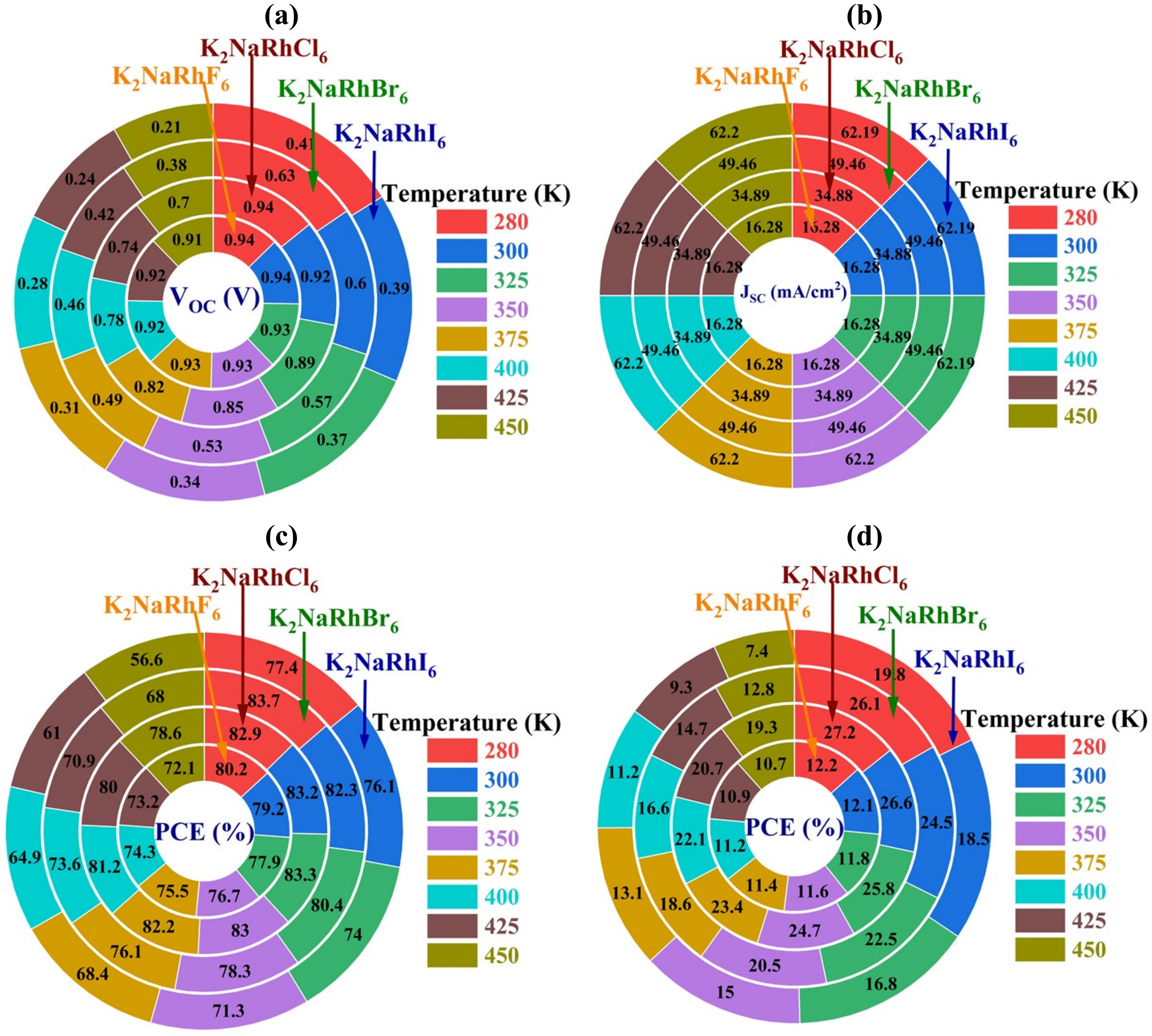

Figure S5. Temperature-dependent photovoltaic performance of K_2NaRhX_6 (X = F, Cl, Br, I) double perovskite solar cells showing variations in **(a)** open-circuit voltage (V_{OC}), **(b)** short-circuit current density (J_{SC}), **(c)** fill factor (FF), and **(d)** power conversion efficiency (PCE) under different operating temperatures.

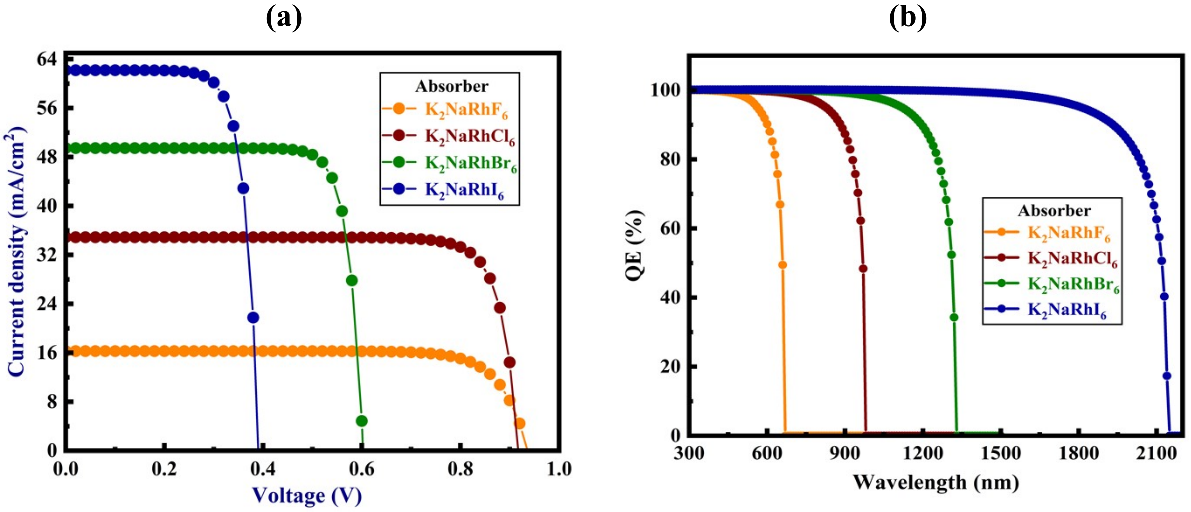

Figure S6. **(a)** Current density-voltage (J-V) characteristics and **(b)** external quantum efficiency (QE) spectra of K_2NaRhX_6 (X = F, Cl, Br, and I) solar cells.

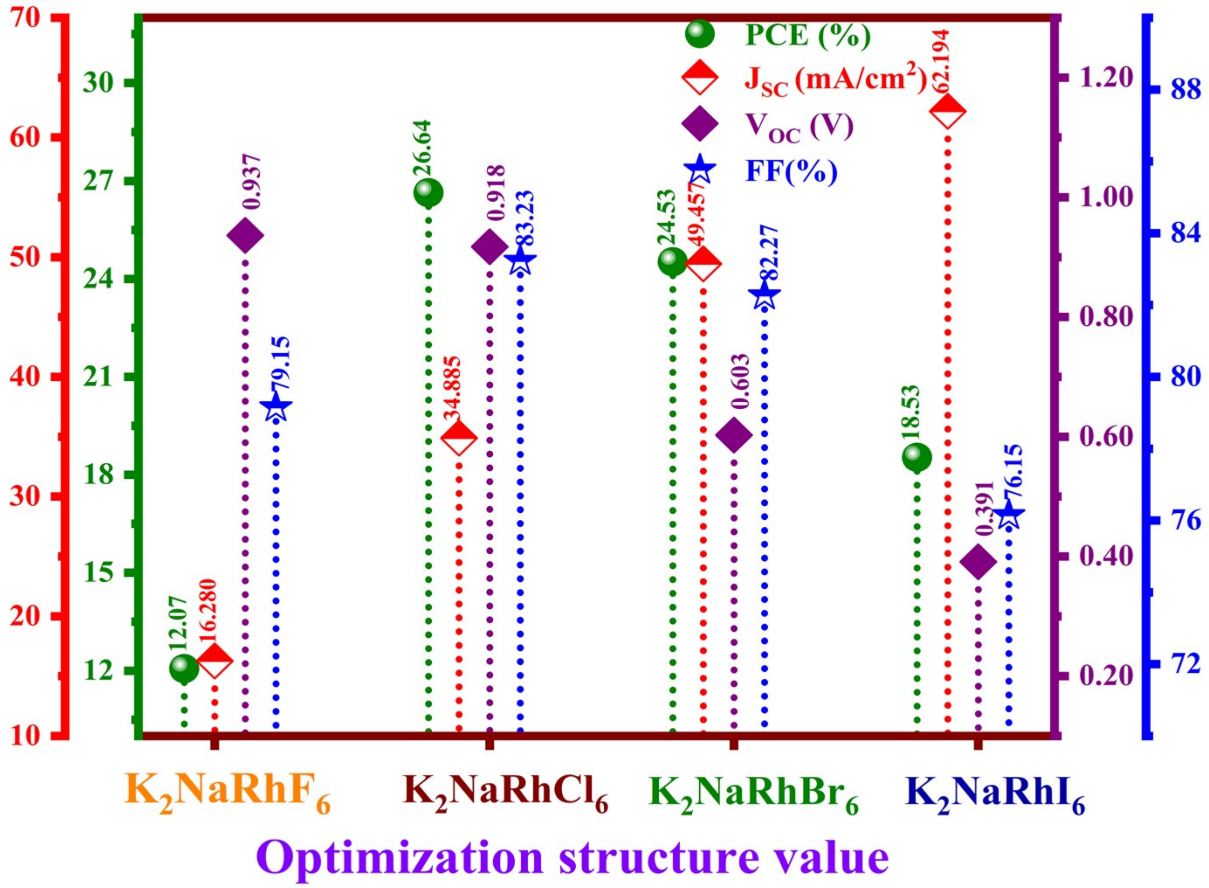

Figure S7. Optimized photovoltaic performance parameters of $CdS/SnS_2/K_2NaRhX_6$ (X = F, Cl, Br, and I) solar cells showing PCE, J_{SC}, V_{OC}, and FF.

References:

1 M. Haghighi, M. Minbashi, N. Taghavinia, D.-H. Kim, S. M. Mahdavi and A. A. Kordbacheh, *Solar Energy*, 2018, **167**, 165–171.

2 H. Bendjilali, S. Rabhi, A. I. Shimul, S. Sadaf, S. A. Ansari and M. W. Alam, *RSC Adv.*, 2026, **16**, 22006–22020.

3 M. Li, S. Guo, X. Zhao, S. Quan, X. Wang, M. Wu, R. Liu and D. Weller, *ACS Appl. Electron. Mater.*, 2024, **6**, 5997–6004.
